# Comparison of Eating Habits, Body Composition and Densitometric Parameters between Subjects with Normal Cognitive Function and Mild Cognitive Impairment: An Observational Study

**DOI:** 10.3390/nu16050644

**Published:** 2024-02-25

**Authors:** Małgorzata Jamka, Maria Chrobot, Natalia Jaworska, Jan Brylak, Aleksandra Makarewicz-Bukowska, Joanna Popek, Adrianna Janicka, Jarosław Walkowiak

**Affiliations:** Department of Pediatric Gastroenterology and Metabolic Diseases, Poznan University of Medical Sciences, Szpitalna Str. 27/33, 60-572 Poznań, Poland; mjamka@ump.edu.pl (M.J.); m.chrobot@ump.edu.pl (M.C.); njaworska@ump.edu.pl (N.J.); j.brylak@ump.edu.pl (J.B.); amakarewicz@ump.edu.pl (A.M.-B.); j.popek@ump.edu.pl (J.P.); adawanot@wp.pl (A.J.)

**Keywords:** dietary habits, dietary intake, body fat distribution, bone mineral density, bone mineral content, cognition

## Abstract

The role of nutrition in the ageing process of the brain is pivotal. Therefore, the study aimed to compare eating habits, body composition and densitometric parameters between subjects with normal cognitive function (NCF) and mild cognitive impairment (MCI). A total of 95 subjects with NCF (74% of women) and 95 individuals with MCI (77% of women) aged 50–70 years were studied. Densitometric parameters were evaluated using dual-energy X-ray absorptiometry methods. Eating habits were assessed using the food frequency questionnaire and 3-day diary records, and advanced glycation end products (AGEs) intake was calculated. Significant differences between groups were detected for the %fat in the right arm (NCF vs. MCI: 38.4 (30.4–46.8) vs. 43.5 (35.5–49.2)%, *p* = 0.0407). Moreover, the MCI group had a significantly lower intake of calcium (*p* = 0.0010), phosphor (*p* = 0.0411), vitamins B2 (*p* = 0.0138) and B12 (*p* = 0.0024) compared to the NCF group, with both groups also differing in the frequency of butter (*p* = 0.0191) and fermented milk beverages (*p* = 0.0398) intake. Analysis restricted to women showed significant differences between groups in right arm %fat, VAT mass, calcium, vitamins B2, B12, butter and fermented milk products intake, while in men, differences were detected in the intake of calcium, iodine, vitamin B1, water and AGEs. In conclusion, subjects with NCF and MCI have comparable densitometric variables but differ significantly in some body composition parameters and the intake of some food groups and nutrients.

## 1. Introduction

Mild cognitive impairment (MCI) is an intermediate condition between normal cognitive function (NCF) and dementia. Many different definitions and diagnostic criteria for diagnosing MCI have been described in the literature. Typically, it is assumed that MCI is characterised by mild memory impairment, which does not, however, disturb the subject’s normal functioning [1]. It is estimated that MCI affects up to 15% of the global population aged 50 and over [2]. These individuals with MCI are at increased risk of developing dementia, with an annual risk of dementia of 10–15% compared to 1–2% for similarly aged people with NCF. Early diagnosis and therapy of MCI may postpone or prevent the development of dementia [3].

Eating habits and nutrition status are important modifiable risk factors for the development of MCI and dementia, as both may play an essential role in the ageing process [4]. It has been suggested that higher consumption of vegetables and fruits [5], fish [6] and nuts [7] is associated with better cognitive functions and a lower risk of developing dementia and that meat consumption may increase the risk of cognitive impairment [8]. The role of nutrients such as fatty acids, vitamins and minerals in developing cognitive disorders has also been investigated, but the results so far have been inconclusive. Nevertheless, some studies suggested that antioxidants, unsaturated fatty acids or some B vitamins may be protective [9,10,11,12]. It has also been suggested that advanced glycation end products (AGEs) intake may affect cognitive function. AGEs are formed mainly during the Maillard reaction [13], and higher levels of AGEs in the brains of subjects with Alzheimer’s disease contribute to amyloid plaque deposition [14]. Moreover, higher AGEs concentrations in blood [15] and urine [16] were associated with more significant cognitive decline. However, only a few studies have evaluated AGEs intake in subjects with cognitive decline, suggesting that higher intake may be associated with faster cognitive impairment [17].

Nutritional status may also determine the prevalence of cognitive impairment, with some findings suggesting that loss of free-fat mass may be linked to cognitive decline [18]. However, the association between body composition and overall cognitive function is controversial, as other studies reported no associations between body composition and cognitive dysfunction [19]. Moreover, a previous meta-analysis showed that obesity and a higher body mass index (BMI) may be associated with an increased risk of dementia [20]. Some data also suggested that bone mineral density (BMD) is reduced in cognitively impaired individuals [21,22], but this has not been confirmed in other studies [23]. Furthermore, the underlying mechanisms for the association between cognition and body composition and densitometric parameters are not yet fully understood.

Therefore, the primary aim of this study was to compare eating habits and nutritional value of diet and AGEs intake between subjects with NCF and MCI. The secondary objective included the evaluation of body composition and densitometric variables in MCI and NCF individuals. Moreover, we also performed separate analyses for men and women.

## 2. Materials and Methods

### 2.1. Study Design and Ethics Issue

This observational study was conducted according to the Strengthening the Reporting of Observational Studies in Epidemiology (STROBE; see Table S1, Supplementary Materials) [24] and the Declaration of Helsinki [25]. The study protocol was approved by the Ethical Committee of the Poznan University of Medical Sciences (protocol no.: 47/20, date of approval: 16 January 2020, with amendments), and all participants provided written informed consent.

### 2.2. Study Population

Participants were recruited from the Greater Poland Voivodeship from July 2021 to August 2022 by physicians at the Department of Pediatric Gastroenterology and Metabolic Diseases, Poznan University of Medical Sciences. The inclusion criteria were age 50–70, Montreal Cognitive Assessment (MOCA) scores of 19–26 points (MCI group) and 27–30 points (NCF group), residing within the community. The exclusion criteria were MOCA scores < 19 points, history of depression treatment and/or Hamilton Depression Rating scale (HAM-D) test scores > 13 points, usage of cognitive enhancement drugs or psychotropic medications, excessive alcohol consumption (>15 units per week), substance abuse disorders, mental health conditions, Parkinson’s disease, Alzheimer’s disease, dementia, all types of anaemia, diabetes ≥ 10 years, severe chronic kidney and liver diseases, a previous cancer diagnosis with chemotherapy or radiotherapy within the last five years, a history of stroke, seizures in the past two years, a head injury leading to loss of consciousness or immediate post-injury confusion, hypothyroidism with current abnormal levels of thyrotropic hormone, any other severe chronic illnesses preventing participation in the study, high levels of physical activity, presence of implanted pacemakers, neurostimulators or other metallic components, including prosthetic implants, blindness, deafness, communication challenges or any other disabilities, which may hinder participation in the study.

### 2.3. Procedures

During the recruitment visit, the physician completed the MOCA and HAM-D questionnaire with potential participants, collected medical information and measured body weight and height. Participants also received a food frequency questionnaire and a 3-day food diary to complete at home for submission at the next visit, during which body composition and densitometric parameters were determined. All measurements were performed at the Department of Pediatric Gastroenterology and Metabolic Diseases, Poznan University of Medical Sciences.

### 2.4. Montreal Cognitive Assessment Scale

The MOCA questionnaire was used to allocate study participants to the MCI and NCF groups and evaluate visuospatial and executive function, naming, memory, attention, language, abstraction, delayed recall and orientation. A qualified physician, appropriately trained and certified for MOCA administration and scoring, conducted the assessment. MOCA scores within the range of 27–30 points are indicative of NCF, while scores within the range of 19–26 points suggest MCI, and scores below 19 points typically lead to a diagnosis of dementia [26].

### 2.5. Hamilton Depression Rating Scale 

The HAM-D questionnaire was used to assess the occurrence of depressive symptoms, with scores of ≥23 indicative of very severe depression and the range of 18–22 signifying severe depression. Scores within the range of 14–18 indicate moderate depression, while the range of 8–13 indicates mild depression, and <7 denotes an absence of depression [27,28].

### 2.6. Anthropometric Parameters

Body weight was measured using an electronic scale with an altimeter (Radwag, WPT 100/200 OW, Radom, Poland) and was performed without shoes and in underwear with an accuracy of 0.1 kg. Body height was measured with an accuracy of 0.5 cm. BMI was calculated to assess the nutritional status of the study population according to the World Health Organization (WHO) criteria. Malnutrition was defined as a BMI of ≤18.5 kg/m^2^; 18.5–24.9 kg/m^2^ was considered within the normal weight range; overweight was classified as a BMI between 25 and 29.9 kg/m^2^; and obesity was represented by a BMI of ≥30 kg/m^2^ [29]. 

### 2.7. Body Composition

Body composition analysis was assessed with dual-energy X-ray absorptiometry (DEXA) methods using the Hologic Discovery analyser (Bedford, MA, USA). The measurement included determining the percentage of body fat (%BF) in the total body and individual areas, such as arms, legs, trunk, male (android) and female (gynoid). Moreover, the visceral adipose tissue (VAT) content was determined, and the proportion of android to visceral fat distribution, as well as the trunk/leg index and fat mass index (FMI), were also calculated. The appendicular lean mass index (ALMI) and lean mass index (LMI) were used to determine the muscle mass content [30].

### 2.8. Densitometric Parameters

Bone mineral content (BMC) and BMD at the lumbar spine (L1–L4) were analysed by DEXA using the Hologic Discovery DXA system (Bedford, MA, USA). All assessments were performed based on the International Society for Clinical Densitometry guidelines [31], with participants in their underwear and without shoes. All metal elements were removed before the measurement. The WHO criteria were used to assess bone health status, with a T-score > −1 indicative of normal bone health, ≤−1 but >−2.5 indicating osteopenia and a T-score ≤ −2.5 suggesting osteoporosis [32].

### 2.9. Eating Habits

A self-administrative version of the Dietary Habits and Nutrition Beliefs Questionnaire (KomPAN, The Committee on Human Nutrition Science, Polish Academy of Sciences, Poland) was used to assess participants’ dietary habits. In our study, part B of the KomPAN questionnaire related to food frequency consumption was administered [33], with nutritional habits also evaluated using diary records. Participants recorded their diet in the diary for three days within one week, including one weekend day, which could be a consecutive or non-consecutive day. A qualified dietician instructed participants on how to complete the questionnaires and checked both questionnaires. The intake of energy, macro- (fats, proteins, carbohydrates) and selected micronutrients (including fatty acids, vitamins and minerals) was calculated using the Aliant software version no: 81 (Anmarsoft, Gdańsk, Poland) based on the 3-day diary records. Moreover, AGEs intake was calculated using the Uribarri et al. [34] database, which comprises the most commonly consumed foods and widely employed culinary techniques in the USA. Consequently, not all food items available in Poland were included in the database; therefore, the AGEs content was estimated by referencing similar foods with similar nutrient and ingredient profiles. In instances where the AGEs content of a specific food prepared with a particular culinary method was unavailable, the AGEs content of a comparable food prepared using a similar culinary method was utilised.

### 2.10. Minimum Sample Size Calculation

The minimum sample size was calculated as 75 subjects per group using the G*Power 3.1 software (University of Kiel, Kiel, Germany) to obtain a power of 80% (α = 0.05, β = 0.2). Considering a maximum dropout rate of 20%, each group should contain at least 90 subjects. This calculation was based on the estimated differences in AGEs intake between groups, as determined in our preliminary study. We assumed that the differences in AGEs intake between the groups would amount to 1500 kU, with a standard deviation of 3250 kU.

### 2.11. Statistical Analysis

Statistics were performed in the Statistica 13.0 program (TIBCO Software Inc., Palo Alto, CA, USA), with a *p*-value < 0.05 considered statistically significant. The normality of the distribution of variables was verified by the Shapiro–Wilk test. Due to the lack of normal distribution for most of the analysed variables, the characteristics of the study population were presented as median and interquartile range (IQR) or in the form of frequencies and percentages. The Mann–Whitney test was used for comparisons with unpaired groups; the Chi^2^ test was used to evaluate categorical variables; and the Spearman index was used to assess the correlation between selected parameters.

## 3. Results

### 3.1. Recruitment Process

**Figure 1:**
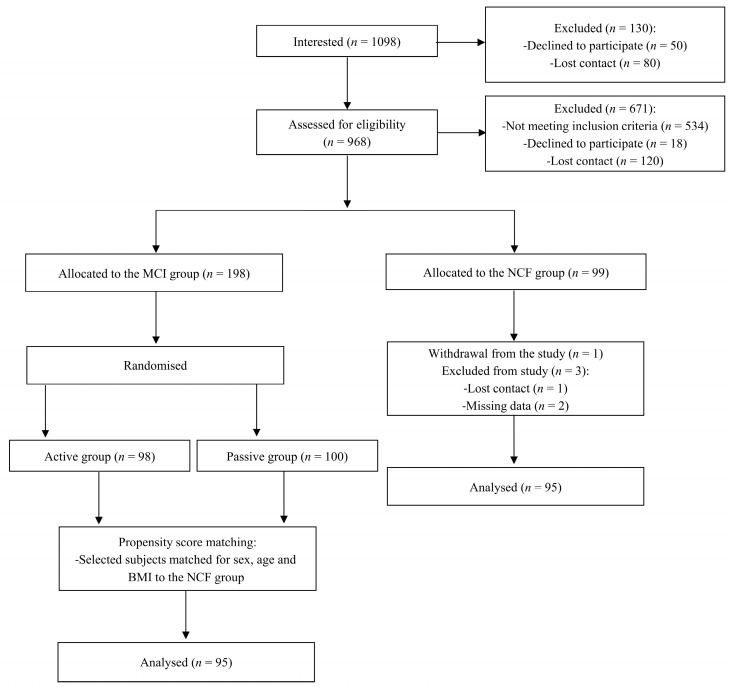
Study flow chart.

Figure 1 depicts the work’s flow. Over 1000 individuals expressed their interest in study participation, of whom 969 subjects were invited to the recruitment visit, and 671 subjects did not meet the inclusion criteria, withdrew from the study or were excluded due to loss of contact. Ultimately, 99 people were included in the NCF group, but 4 individuals dropped out of the study. Therefore, the final NCF group consisted of 95 participants. The MCI group also included 95 subjects. Subjects in the MCI group were selected from among 198 people who took part in the randomised controlled trial [35]. The MCI group was matched with the NCF group in terms of age, sex and BMI. The baseline characteristics of the study population are presented in Table 1, with no differences between groups regarding sex, age, body weight, BMI and HAM-D. Separate results for men and women are presented in Table S2 (see Supplementary Materials).

### 3.2. Comparison of Body Composition between Subjects with Normal Cognitive Function and Mild Cognitive Impairment

Table 2 presents the comparison of body composition between subjects with NCF and MCI. Significant differences between groups were detected for the %BF in the right arm (*p* = 0.0407), with lower values observed in the NCFgroup. However, when we performed separate analyses for men and women, this tendency was seen only in the women group (*p* = 0.0456). Moreover, women with MCI had significantly higher VAT mass than women with NCF (*p* = 0.0487; see Table S3, Supplementary Materials).

### 3.3. Comparison of Densitometric Parameters between Subjects with Normal Cognitive Function and Mild Cognitive Impairment

A comparison of the groups’ densitometric parameters is provided in Table 3 and Table S4, showing no differences in BMC, BMD, T-score and Z-score at the lumbar spine (L1–L4) between subjects with NCF and MCI.

### 3.4. Comparison of Intake of Energy and Selected Macro- and Micronutrients between Subjects with Normal Cognitive Function and Mild Cognitive Impairment

Table 4 presents a comparison of the nutritional value of the subjects’ diets. Significant differences between groups were detected in the intake of calcium (*p =* 0.0010), phosphor (*p =* 0.0411), vitamin B2 (*p =* 0.0138) and vitamin B12 (*p =* 0.0024), but no differences in AGEs intake were noted. Separate analyses for women and men are shown in Table S5 (see Supplementary Materials). Women with MCI significantly differed from women with NCF in the intake of calcium (*p* = 0.0195), vitamin B2 (*p* = 0.0495) and vitamin B12 (*p =* 0.0159). In the men group, differences between groups were found in the intake of calcium (*p* = 0.0165), iodine (*p* = 0.0485), vitamin B1 (*p* = 0.0180), water (*p* = 0.0024) and AGEs (*p* = 0.0244).

### 3.5. Comparison of the Frequency of Consumption of Selected Food Products between Subjects with Normal Cognitive Function and Mild Cognitive Impairment

A comparison of the frequency of consumption of selected food products between subjects with NCF and MCI is included in Table 5, with both groups differing only in butter (*p* = 0.0191) and fermented milk beverages (*p* = 0.0398) intake. When we performed separate analyses for men and women, significant differences in the frequency of intake of butter (*p* = 0.0173) and fermented milk beverages (*p* = 0.0235) between MCI and NCF subjects were observed only in the women group (see Table S6, Supplementary Materials).

### 3.6. Correlations between Montreal Cognitive Assessment Scale Results and Selected Variables

An inverse correlation between vitamin B12 and MOCA results was observed (*rho* = −0.2161, *p* = 0.0354) in the NCF group. In the MCI group, the MOCA results were negatively correlated with diet energy value (*rho* = −0.2597, *p* = 0.0110), fat intake (*rho* = −0.2083, *p* = 0.0428), digestible carbohydrate intake (*rho* = −0.2215, *p* = 0.0310) and saturated fatty acids intake (*rho* = −0.2157, *p* = 0.0358), while a positive correlation was found with the percentage of energy from protein (*rho* = 0.2356, *p* = 0.0216). 

## 4. Discussion

There were no differences in densitometric parameters between subjects with NCF and MCI. However, some differences between groups in terms of body composition parameters and the nutritional value of diet and some food product intakes were noted.

Previously, it has been suggested that cognitive decline may affect the bone remodelling process [36]. Indeed, Zhang et al. [21] showed a significant decrease in BMD in subjects with Alzheimer’s disease compared to NCF participants and a positive correlation between BMD and scores obtained in the Minimal Mental State Examination (MMSE) scale. In addition, the receiver operator characteristic curve analysis indicated that this densitometric variable could be used to distinguish cognitive impairment participants from NCF individuals. Similar results were obtained by Lee et al. [22], who reported that cognitive impairment was associated with lower BMD at the lumbar spine and total hip. However, patients with Alzheimer’s disease were compared to subjects with subjective cognitive impairment in this study. Additionally, Lin et al. [37] reported that BMD is effective in predicting MMSE scores. Furthermore, Noh et al. [38] showed that a higher BMC at the arm was associated with a decreased probability of MCI development, but this association was no longer significant after adjusting for potential confounding factors. In contrast, Patel et al. [23] observed no association between cognitive function and densitometric markers, which is in line with our results. No association between cognition and bone parameters was also found by Nourhashemi et al. [39]. We hypothesise that the differences between the study results may be due to differences in the age and sex of the study participants.

It is also speculated that changes in body composition might be associated with cognitive decline. To date, several studies reported that lower free-fat mass is related to a higher risk of developing MCI [18,39]. A decrease in lean mass is generally observed with ageing and is frequently associated with low diet quality [40] and low physical activity [41], both of which are also common in cognitive decline. Other mechanisms involved in this process may be associated with oxidative stress, the inflammatory process and hormonal changes [39]. However, some studies suggested that an increase in %BF may be associated with better cognitive functions [42]. It is assumed that the higher concentrations of leptin observed in subjects with higher fat tissue content may be responsible for the protective effect of preventing cognitive disorders [43]. Moreover, a higher %BF is often related to a higher BMI, while BMI is positively correlated with white matter volume [44]. In contrast, another study demonstrated a lack of association between body composition and cognition, but due to the small sample size, the statistical power of this study was low [19]. Differences in the results in selected studies may be due to the use of different tools to assess cognitive function and measure body composition or differences in subjects’ race or ethnicity. In our study, significant differences between groups were detected only for %BF in the right arm, with lower values found in the NCF group. Notably, a subgroup analysis confirmed these differences only in the women group. Moreover, women in the MCI group also had a significantly higher VAT mass than women in the NCF group. The underlying mechanism for the observed disparity in %BF in the right arm remains unclear. We hypothesise that this may be linked to the handedness of participants, although, due to the lack of data on their dominant hands, this remains speculative. The observed differences between the MCI and NCF groups in body composition parameters may also be associated with higher physical activity in subjects with NCF compared to MCI individuals. Indeed, our previous study showed that NCF participants, compared to people with MCI, are characterised by higher total and moderate physical activity and lower sedentary activity measured by the ActiGraph [41]. 

Some nutrients, such as antioxidants, B vitamins or unsaturated fatty acids, could potentially have significant impacts on brain function [45,46]. Therefore, it is suggested that the intake of some nutrients may play an important role in preventing cognitive disorders. To date, several studies have compared the eating habits of subjects with MCI with the eating habits of individuals with NCF, providing unequivocal results. In our study, significant differences between MCI and NCF groups were detected in the intake of calcium, phosphor, vitamin B2 and vitamin B12, with lower intake observed in MCI individuals. Differences between groups in the intake of calcium, vitamin B2 and vitamin B12 were confirmed in a separate analysis for women. In addition, analysis restricted to men also showed significant differences between groups in the intake of calcium, iodine, vitamin B1 and water. Indeed, previous findings suggested that B vitamins might modulate the prevalence of cognitive decline. It is well known that vitamin B12 is involved in the DNA methylation process and the conversion of homocysteine to methionine, while higher levels of homocysteine may potentially result in a neurotoxic effect [47]. As higher concentrations of homocysteine were noted in subjects with dementia, it was speculated that homocysteine levels may predict the risk of development of cognitive decline [48]. In addition, a higher vitamin B2 intake was noted in subjects with higher MMSE scores by Requejo et al. [49]. Moreover, Ozawa et al. [50] observed that higher self-reported intake of some minerals, such as potassium, calcium and magnesium, was associated with a lower risk of developing cognitive impairment. These findings are partly in line with our results, as we found a low intake of calcium and phosphorus in subjects with MCI. In contrast, Cherbuin et al. [51] demonstrated that higher potassium and iron consumption increased the risk of developing MCI. The mechanism through which the risk of cognitive decline changes with mineral intake is unclear, but it is suggested that, for potassium, this could be associated with an antihypertension effect [52]. Additionally, several studies reported the protective effects of dietary antioxidants on cognition [49,52], but we did not observe any differences in the intake of antioxidant vitamins between subjects with NCF and MCI. Similarly, no differences in fatty acid intake were observed between groups, while previous results suggested that the intake of unsaturated fats, especially monounsaturated fatty acids and n-3 polyunsaturated fatty acids, might protect against cognitive decline [11]. In addition, there were no differences between groups in the present study regarding the intake of calories, fats, proteins and carbohydrates, while some previous studies suggested that diet macronutrient distribution might affect cognitive function [53]. In contrast, similar to our study, other studies did not demonstrate differences in energy or macronutrient intake between subjects with Alzheimer’s disease, MCI and controls [54]. We speculate that potential differences between the studies’ results may be related to different dietary assessment methods. Additionally, current intake may not reflect the intake, which has occurred over the past years.

Previously, higher AGEs levels were associated with greater cognitive decline through the effects on β-amyloid and tau protein metabolism [16]. Moreover, Fleitas et al. [55] postulated that AGEs may modify the precursor form of brain-derived neurotrophic factor, leading to neuronal apoptosis by inducing the processing of the p75 neurotrophic receptor. Therefore, we hypothesised that MCI and NCF subjects might differ significantly in AGEs intake, but our results did not confirm this hypothesis, as we noted no differences between the groups. However, a separate analysis for men showed that MCI subjects intake significantly higher amounts of AGEs than NCF individuals. Moreover, the calculated AGEs intake in the present study was similar to the results reported among healthy subjects [56]. Nevertheless, West et al. [17] showed that higher dietary AGEs intake was associated with faster cognitive decline. Moreover, Lotan et al. [57] found that a decrease in AGE intake improves cognitive function in subjects with diabetes. Due to unequivocal results, further studies are needed to assess whether subjects with MCI differ from subjects with NCF in AGEs consumption.

Previous studies suggested that healthy eating habits may protect against the development of cognitive impairment. However, Milte et al. [58] showed that diet variety, not quality, was associated with cognitive function. Nevertheless, a potential mechanism by which a healthy diet may protect against cognitive decline is associated with, among other things, a positive effect of diet on the cardiovascular system [59]. Therefore, we hypothesise that subjects with MCI may significantly differ from NCF participants in the frequency of intake of selected food products. However, our study comparing the intake of selected food groups found that MCI and NCF subjects differed only in the frequency of butter and fermented milk beverages intake, with more frequent consumption in the NCF group. However, when we conducted a separate analysis for each sex, these associations were detected only in women. Additionally, Wang et al. [60] demonstrated a higher intake of animal oil in the NCF elderly Chinese subjects compared to MCI participants. Nevertheless, these findings were somewhat surprising, despite a previous meta-analysis reporting that higher milk consumption was associated with a reduced risk of cognitive decline [61]. We rather expected to find significant differences between groups in the frequency of fruit and vegetable intake, as their higher consumption is associated with a lower incidence of cognitive disorders [62]. Okubo et al. [63] also showed that plant and fish food pattern was associated with higher scores obtained in the MOCA test. Moreover, higher adherence to the Mediterranean diet—which is characterised by high consumption of vegetables and fruits, legumes and cereals, moderate-to-high intake of fish and other sources of unsaturated fatty acids, low-to-moderate intake of dairy products, low intake of meat and saturated fatty acids, and a regular but moderate intake of alcohol—is a known protective factor against cognitive disorders [64]. We speculate that our study may have had inadequate power to detect significant differences in the intake of other food groups.

This study’s strengths include strict and clearly defined inclusion and exclusion criteria and the use of propensity score matching to match both groups in terms of age, sex and BMI. Moreover, two methods were used (the KomPAN survey and a 3-day food diary) to determine the eating habits of the study population. Furthermore, this is one of the first studies comparing AGEs intake between subjects with NCF and MCI.

The study’s limitations include the allocation of study participants to the MCI and NCF groups based only on the MOCA test results. Another limitation is using the self-completed version of the KomPAN questionnaire and a 3-day food diary, which may have introduced reporting bias in food intake. However, a qualified dietitian instructed participants on completing the survey and verified whether the study participants had completed both questionnaires correctly. In addition, subjects with MCI may not be able to accurately assess their dietary intake using subjective methods. Indeed, our previous study showed that objective rather than subjective methods are more reliable in assessing physical activity in MCI individuals [41].

Another limitation is that the KomPAN questionnaire is validated only for individuals up to 65 years of age. However, the choice of this questionnaire resulted from the initial study inclusion criteria, which was 50–65 years of age, but due to difficulties in recruiting an adequate number of subjects with MCI, the age criteria were expanded to 50–70 years. In addition, dietary supplement intake was not monitored. Furthermore, it should be noted that the AGEs database utilised in the current study was originally established in the USA, and there are significantly diverse dietary patterns between the USA and Poland. This database exclusively includes carboxymethyl-lysine as an indicator of AGEs, omitting other significant markers, such as carboxyethyl-lysine and methylglyoxal-derived hydroimidazolone 1, and it has a limited number of records.

To sum up, our results showed significant differences between groups in the intake of butter and fermented milk beverages, as well as calcium, phosphor, vitamins B2 and B12. In addition, individuals with NCF had a significantly lower %BF in the right arm compared to the MCI group. Moreover, a separate analysis for women revealed significant differences between groups in %BF in the right arm, VAT mass, calcium, vitamin B2, vitamin B12, butter and fermented milk products intake, while in the men group, differences were detected in the intake of calcium, iodine, vitamin B1, water and AGEs.

## 5. Conclusions

In conclusion, subjects with NCF and MCI did not differ in densitometric variables, but there were significant differences between groups in some body composition parameters, the intake of certain food groups and nutrients. Moreover, differences in eating habits and body composition between the MCI and NCF groups may be dependent on sex. However, the small sample size limited these findings; therefore, further studies are needed to confirm these results.

## Figures and Tables

**Table 1:** Characteristics of subjects with NCF and MCI.

		Median (IQR)/*n* (%)			*p*
		Total (*n* = 190)	NCF (*n* = 95)	MCI (*n* = 95)	
Sex [n (%)]	Women	143 (75.3%)	70 (73.7%)	73 (76.8%)	0.3685
	Men	47 (24.7%)	25 (26.3%)	22 (23.2%)	
Age [years]		56 (52–61)	56 (53–61)	57 (52–61)	0.9294
Weight [kg]		74.00 (63.00–86.80)	73.85 (61.70–90.40)	74.00 (63.00–84.00)	0.6642
Height [m]		1.65 (1.61–1.73)	1.67 (1.62–1.73)	1.64 (1.60–1.72)	0.0636
BMI [kg/m^2^]		26.58 (23.60–29.80)	26.45 (22.94–30.49)	26.64 (24.30–29.58)	0.4426
HAM-D [points]		3 (1–5)	4 (1–6)	3 (1–5)	0.2537

BMI—body mass index; HAM-D—Hamilton Depression Rating scale; IQR—interquartile range; MCI—mild cognitive impairment; NCF—normal cognitive function.

**Table 2:** Comparison of body composition between subjects with NCF and MCI.

			Median (IQR)		*p*
		Total (*n* = 190)	NCF (*n* = 95)	MCI (*n* = 95)	
BF [%]	Left arm	43.2 (31.9–49.0)	41.6 (31.0–48.8)	44.7 (35.1–49.9)	0.1164
	Right arm	41.8 (31.4–47.9)	38.4 (30.4–46.8)	43.5 (35.5–49.2)	0.0407
	Trunk	36 (30.8–40.7)	35.6 (29.3–40.0)	37.3 (31.5–41.5)	0.0798
	Left leg	38.6 (30.6–43.5)	37.1 (28.5–42.5)	39.7 (31.1–43.7)	0.2106
	Right leg	38.5 (30.2–43.7)	37.5 (30.2–42.7)	39.9 (30.2–44.2)	0.2985
	Total	35.9 (31.0–41.0)	35.0 (29.9–40.1)	37.2 (32.4–41.6)	0.0861
	Male (android)	38.1 (32.8–43.0)	37.3 (29.7–42.7)	39.1 (33.8–43.7)	0.0926
	Female (gynoid)	39.0 (31.7–42.8)	37.5 (31.6–42.8)	39.6 (31.7–42.9)	0.2164
FMI [kg/m^2^]		9.0 (7.4–12.1)	8.9 (6.9–11.5)	9.6 (7.6–12.3)	0.1460
Android/gynoid ratio		1.0 (0.9–1.2)	1.0 (0.9–1.2)	1.0 (0.9–1.2)	0.5369
Trunk/leg fat ratio		1.0 (0.9–1.2)	1.0 (0.9–1.1)	1.0 (0.9–1.3)	0.6815
VAT [g]		605.5 (394.0–822.0)	562.0 (343.0–843.0)	637.0 (424.0–809.0)	0.1806
LMI [kg/m^2^]		15.8 (14.1–17.9)	15.6 (14.0–18.0)	15.8 (14.2–17.6)	0.8794
ALMI [kg/m^2^]		6.7 (5.9–7.7)	6.7 (5.9–8.0)	6.7 (5.9–7.6)	0.5171

ALMI—appendicular lean mass index; BF—body fat; FMI—fat mass index; IQR—interquartile range; LMI—lean mass index; MCI—mild cognitive impairment; NCF—normal cognitive function; VAT—visceral adipose tissue.

**Table 3:** Comparison of densitometric parameters between subjects with NCF and MCI.

		Median (IQR)			*p*
		Total (*n* = 189)	NCF (*n* = 95)	MCI (*n* = 94)	
Lumbar spine (L1–L4)	BMC [g]	61.10 (51.66–72.00)	61.11 (51.64–72.02)	61.00 (51.66–70.99)	0.6958
	BMD [g/cm^2^]	1.00 (0.90–1.11)	1.00 (0.88–1.12)	0.99 (0.90–1.10)	0.7067
	Z-score	0.50 (−0.30–1.50)	0.40 (−0.40–1.50)	0.60 (−0.30–1.50)	0.7943
	T-score	−0.60 (−1.50–0.40)	−0.60 (−1.70–0.50)	−0.50 (−1.5–0.30)	0.8077

BMC—bone mineral content; BMD—bone mineral density; IQR—interquartile range; MCI—mild cognitive impairment; NCF—normal cognitive function.

**Table 4:** Comparison of intake of energy and selected macro- and micronutrients between subjects with NCF and MCI.

	Median (IQR)			*p*
	Total (*n* = 190)	NCF (*n* = 95)	MCI (*n* = 95)	
Energy [kcal]	1814 (1543–2176)	1860 (1586–2185)	1765 (1536–2101)	0.2325
Protein [g]	72.3 (60.8–87.1)	73.1 (60.3–94.0)	71.9 (60.8–83.2)	0.2007
Protein [%]	15.8 (14.0–17.7)	15.8 (14.0–17.8)	15.7 (14.0–17.4)	0.5705
Fat [g]	72.6 (57.5–91.6)	73.1 (60.3–94.0)	71.6 (57.5–92.5)	0.7158
Fat [%]	35.4 (31.2–40.1)	35.1 (29.7–40.2)	35.8 (31.4–40.1)	0.2918
Carbohydrate [g]	219.4 (182.3–271.8)	222.4 (185.8–274.7)	210.6 (178.5–269.0)	0.5616
Carbohydrate [%]	46.9 (42.5–51.6)	46.4 (42.9–51.6)	47.1 (41.5–52.0)	0.9118
Digestible carbohydrate [g]	197.5 (164.7–244.9)	203.2 (168.9–253.4)	190.8 (157.9–244.9)	0.4576
Fiber [g]	21.5 (16.6–26.9)	20.5 (16.3–26.6)	22.1 (17.4–28.2)	0.3244
Sugar [g]	70.8 (53.9–96.8)	71.9 (56.0–98.4)	69.6 (52.6–94.6)	0.5078
Sugar [%]	15.8 (12.4–19.8)	16.5 (12.8–20.3)	15.4 (12.2–18.5)	0.5214
SFA [g]	27.5 (19.7–33.6)	28.2 (19.7–34.4)	25.4 (19.7–32.9)	0.2478
SFA [%]	12.9 (11.1–15.5)	13.3 (11.2–15.5)	12.8 (10.7–15.6)	0.4379
MUFA [g]	25.5 (19.5–33.0)	25.5 (18.9–34.9)	25.4 (19.7–32.1)	0.8184
MUFA [%]	12.5 (10.7–14.8)	12.3 (10.5–15.1)	12.9 (10.7–14.7)	0.6088
*n*-3 [g]	1.8 (1.3–2.7)	1.7 (1.3–2.4)	1.8 (1.2–2.9)	0.9779
*n*-3 [%]	0.9 (0.7–1.2)	0.9 (0.7–1.1)	0.9 (0.7–1.2)	0.7515
*n*-6 [g]	8.7 (6.0–11.6)	8.5 (6.0–11.6)	8.8 (6.0–11.8)	0.8143
*n*-6 [%]	4.0 (3.1–5.6)	3.9 (3.1–5.2)	4.1 (3.3–5.6)	0.2289
PUFA [g]	11.0 (7.9–14.4)	10.8 (7.9–14.2)	11.6 (7.8–15.3)	0.5257
PUFA [%]	5.1 (4.0–7.1)	4.7 (3.9–6.8)	5.5 (4.1–7.2)	0.1259
LA [g]	8.7 (6.0–11.5)	8.4 (6.0–11.5)	8.7 (5.8–11.7)	0.8338
LA [%]	3.9 (3.1–5.5)	3.8 (3.0–5.0)	4.1 (3.2–5.6)	0.2468
ALA [g]	1.6 (1.2–2.2)	1.6 (1.1–2.2)	1.6 (1.2–2.3)	0.8409
ALA [%]	0.8 (0.6–1.0)	0.8 (0.6–1.1)	0.8 (0.6–1.0)	0.8000
DHA [g]	0.1 (0.0–0.1)	0.1 (0.0–0.1)	0.1 (0.0–0.1)	0.4665
EPA [g]	0.0 (0.0–0.0)	0.0 (0.0–0.0)	0.0 (0.0–0.1)	0.1522
Cholesterol [mg]	310 (227–397)	320 (229–399)	289 (224–389)	0.6652
Salt [g]	4.9 (3.7–6.8)	4.8 (3.9–7.2)	4.9 (3.6–6.5)	0.6142
Sodium [mg]	1890.0 (1446.2–2630.5)	1862.9 (1521.8–2805.7)	1910.4 (1389.1–2507.4)	0.6292
Potassium [mg]	3012.0 (2536.3–3647.4)	3084.9 (2517.7–3647.4)	2964.1 (2536.3–3649.1)	0.6672
Calcium [mg]	648.1 (480.0–808.8)	719.7 (571.9–857.2)	574.0 (441.9–753.7)	0.0010
Phosphor [mg]	1131.9 (969.2–1403.2)	1157.4 (1015.5–1430.7)	1083.1 (901.1–1371.0)	0.0411
Magnesium [mg]	294.4 (242.4–358.7)	286.6 (243.2–349.9)	301.2 (237.8–363.9)	0.9285
Iron [mg]	10.9 (9.1–13.4)	11.0 (9.5–13.4)	10.7 (8.4–13.6)	0.3186
Zinc [mg]	9.3 (7.4–11.3)	9.6 (7.7–11.5)	9.0 (7.1–11.0)	0.1384
Copper [mg]	1.2 (1.0–1.6)	1.2 (1.0–1.6)	1.3 (1.0–1.6)	0.9578
Manganese [mg]	4.3 (3.0–5.7)	4.3 (3.2–5.8)	4.3 (2.9–5.6)	0.6489
Selenium [µg]	5.4 (2.5–14.7)	5.9 (3.1–14.1)	4.5 (2.1–15.5)	0.1717
Iodine [µg]	35.8 (25.4–55.8)	35.8 (26.8–57.2)	35.8 (22.7–52.8)	0.3662
Vit. A [µg]	962.9 (716.6–1336.4)	961.4 (709.2–1395.4)	964.3 (716.6–1327.4)	0.8144
Retinol [µg]	357.3 (235.8–502.1)	380.8 (264.6–577.0)	340.8 (214.3–484.1)	0.1453
β-carotene [µg]	3040.9 (1818.6–4935.3)	3041.6 (1656.9–4935.3)	3024.5 (1985.1–4942.5)	0.4195
Vit. D [µg]	2.0 (1.5–3.1)	1.9 (1.4–3.1)	2.1 (1.6–3.2)	0.2791
Vit. E [mg]	10.4 (8.0–13.4)	10.1 (8.0–13.4)	10.8 (8.2–13.4)	0.5669
Vit. K [µg]	12.5 (4.1–46.0)	12.5 (4.5–51.5)	12.5 (4.0–32.7)	0.6264
Vit. B1 [mg]	1.2 (0.9–1.4)	1.2 (0.9–1.4)	1.1 (0.9–1.5)	0.4810
Vit. B2 [mg]	1.7 (1.4–2.1)	1.8 (1.5–2.2)	1.7 (1.3–2.0)	0.0138
Vit. B3 [mg]	15.7 (11.4–19.2)	15.1 (11.5–19.0)	15.8 (10.8–19.3)	0.9306
Vit. B6 [mg]	1.7 (1.3–2.0)	1.6 (1.3–2.1)	1.7 (1.3–2.0)	0.6579
Folates [µg]	326.1 (268.6–388.2)	333.5 (268.4–392.0)	313.9 (268.6–383.8)	0.4811
Vit. B12 [µg]	2.9 (2.1–4.1)	3.1 (2.5–4.6)	2.7 (1.9–3.5)	0.0024
Vit. C [mg]	141.1 (96.0–192.1)	145.8 (96.7–191.3)	136.8 (95.2–192.1)	0.5240
Water [g]	2078.5 (1545.6–2550.5)	2222.5 (1568.8–2559.3)	1993.1 (1522.9–2527.9)	0.6576
AGEs [kU]	9877.5 (6934.2–13,983.4)	9743.4 (6854.3–13,680.5)	9964.8 (6934.2–15,317.1)	0.6414

AGEs—advanced glycation end products; ALA—α-linolenic acid; DHA—docosahexaenoic acid; EPA—eicosapentaenoic acid; IQR—interquartile range; LA—linoleic acid; MCI—mild cognitive impairment; MUFA—monounsaturated fatty acids; NCF—normal cognitive function; PUFA—polyunsaturated fatty acids; SFA—saturated fatty acids; Vit.—vitamin.

**Table 5:** Comparison of frequency of consumption of selected food products between subjects with NCF and MCI.

		*n* (%)			*p*
		Total (*n* = 190)	NCF (*n* = 95)	MCI (*n* = 95)	
White bread	Never	8 (4.2%)	4 (4.2%)	4 (4.2%)	0.5570
	1–3 times a month	33 (17.4%)	13 (13.7%)	20 (21.1%)	
	Once a week	20 (10.5%)	9 (9.5%)	11 (11.5%)	
	Several times a week	44 (23.2%)	25 (26.3%)	19 (20.0%)	
	Once a day	36 (18.9%)	16 (16.8%)	20 (21.1%)	
	Several times a day	49 (25.8%)	28 (29.5%)	21 (22.1%)	
Wholemeal bread	Never	17 (9.0%)	7 (7.4%)	10 (10.5%)	0.6024
	1–3 times a month	37 (19.5%)	20 (21.1%)	17 (18.0%)	
	Once a week	20 (10.5%)	8 (8.4%)	12 (12.6%)	
	Several times a week	71 (37.3%)	40 (42.1%)	31 (32.6%)	
	Once a day	27 (14.2%)	11 (11.5%)	16 (16.8%)	
	Several times a day	18 (9.5%)	9 (9.5%)	9 (9.5%)	
White rice, pasta or small grains	Never	6 (3.2%)	1 (1.1%)	5 (5.3%)	0.2538
	1–3 times a month	65 (34.2%)	33 (34.7%)	32 (33.7%)	
	Once a week	52 (27.4%)	23 (24.2%)	29 (30.5%)	
	Several times a week	66 (34.7%)	37 (38.9%)	29 (30.5%)	
	Once a day	1 (0.5%)	1 (1.1%)	0 (0.0%)	
	Several times a day	0 (0.0%)	0 (0.0%)	0 (0.0%)	
Buckwheat, oatmeal, whole-wheat pasta or other coarse-grain cereals	Never	19 (10.0%)	7 (7.4%)	12 (12.6%)	0.8000
	1–3 times a month	68 (35.8%)	36 (38.0%)	32 (33.7%)	
	Once a week	29 (15.3%)	14 (14.7%)	15 (15.8%)	
	Several times a week	51 (26.8%)	26 (27.3%)	25 (26.3%)	
	Once a day	23 (12.1%)	12 (12.6%)	11 (11.6%)	
	Several times a day	0 (0.0%)	0 (0.0%)	0 (0.0%)	
Fast foods	Never	51 (26.8%)	24 (25.3%)	27 (28.4%)	0.6799
	1–3 times a month	131 (69.0%)	68 (71.5%)	63 (66.3%)	
	Once a week	7 (3.7%)	3 (3.2%)	4 (4.2%)	
	Several times a week	1 (0.5%)	0 (0%)	1 (1.1%)	
	Once a day	0 (0.0%)	0 (0.0%)	0 (0.0%)	
	Several times a day	0 (0.0%)	0 (0.0%)	0 (0.0%)	
Fried foods	Never	7 (3.7%)	0 (0.0%)	7 (7.4%)	0.0674
	1–3 times a month	58 (30.5%)	28 (29.5%)	30 (31.6%)	
	Once a week	48 (25.3%)	26 (27.3%)	22 (23.1%)	
	Several times a week	71 (37.3%)	39 (41.1%)	32 (33.7%)	
	Once a day	6 (3.2%)	2 (2.1%)	4 (4.2%)	
	Several times a day	0 (0.0%)	0 (0.0%)	0 (0.0%)	
Butter	Never	15 (7.9%)	4 (4.2%)	11 (11.6%)	0.0191
	1–3 times a month	24 (12.6%)	13 (13.7%)	11 (11.6%)	
	Once a week	17 (9.0%)	5 (5.3%)	12 (12.6%)	
	Several times a week	36 (18.9%)	17 (17.9%)	19 (20.0%)	
	Once a day	50 (26.3%)	23 (24.2%)	27 (28.4%)	
	Several times a day	48 (25.3%)	33 (34.7%)	15 (15.8%)	
Lard ^1^	Never	128 (67.7%)	65 (69.1%)	63 (66.3%)	0.5191
	1–3 times a month	52 (27.5%)	27 (28.7%)	25 (26.3%)	
	Once a week	5 (2.7%)	1 (1.1%)	4 (4.2%)	
	Several times a week	3 (1.6%)	1 (1.1%)	2 (2.1%)	
	Once a day	1 (0.5%)	0 (0.0%)	1 (1.1%)	
	Several times a day	0 (0.0%)	0 (0.0%)	0 (0.0%)	
Oils or margarines ^1^	Never	45 (23.8%)	18 (19.1%)	27 (28.5%)	0.0776
	1–3 times a month	24 (12.7%)	12 (12.7%)	12 (12.6%)	
	Once a week	19 (10.1%)	9 (9.6%)	10 (10.5%)	
	Several times a week	62 (32.8%)	40 (42.6%)	22 (23.2%)	
	Once a day	28 (14.8%)	12 (12.8%)	16 (16.8%)	
	Several times a day	11 (5.8%)	3 (3.2%)	8 (8.4%)	
Milk	Never	36 (19.0%)	16 (16.9%)	20 (21.1%)	0.9463
	1–3 times a month	30 (15.8%)	14 (14.7%)	16 (16.8%)	
	Once a week	13 (6.8%)	6 (6.3%)	7 (7.3%)	
	Several times a week	29 (15.3%)	16 (16.8%)	13 (13.7%)	
	Once a day	47 (24.7%)	25 (26.3%)	22 (23.2%)	
	Several times a day	35 (18.4%)	18 (19.0%)	17 (17.9%)	
Fermented milk beverages	Never	5 (2.6%)	1 (1.0%)	4 (4.2%)	0.0398
	1–3 times a month	32 (16.9%)	15 (15.8%)	17 (17.9%)	
	Once a week	31 (16.3%)	21 (22.1%)	10 (10.5%)	
	Several times a week	95 (50.0%)	40 (42.1%)	55 (57.9%)	
	Once a day	26 (13.7%)	17 (18.0%)	9 (9.5%)	
	Several times a day	1 (0.5%)	1 (1.0%)	0 (0.0%)	
Quark	Never	8 (4.2%)	2 (2.11%)	6 (6.3%)	0.3984
	1–3 times a month	32 (16.8%)	12 (12.63%)	20 (21.1%)	
	Once a week	43 (22.6%)	23 (24.21%)	20 (21.1%)	
	Several times a week	72 (38.0%)	38 (40.0%)	34 (35.8%)	
	Once a day	28 (14.7%)	16 (16.88%)	12 (12.6%)	
	Several times a day	7 (3.7%)	4 (4.2%)	3 (3.2%)	
Cheese	Never	6 (3.2%)	4 (4.2%)	2 (2.1%)	0.1748
	1–3 times a month	35 (18.4%)	13 (13.7%)	22 (23.2%)	
	Once a week	47 (24.7%)	24 (25.2%)	23 (24.2%)	
	Several times a week	76 (40.0%)	36 (37.9%)	40 (42.1%)	
	Once a day	20 (10.5%)	13 (13.7%)	7 (7.3%)	
	Several times a day	6 (3.2%)	5 (5.3%)	1 (1.1%)	
Meats or sausages	Never	11 (5.8%)	6 (6.3%)	5 (5.3%)	0.5542
	1–3 times a month	24 (12.6%)	11 (11.6%)	13 (13.7%)	
	Once a week	25 (13.2%)	10 (10.5%)	15 (15.8%)	
	Several times a week	94 (49.5%)	46 (48.5%)	48 (50.5%)	
	Once a day	24 (12.6%)	16 (16.8%)	8 (8.4%)	
	Several times a day	12 (6.3%)	6 (6.3%)	6 (6.3%)	
Red meat	Never	19 (10.0%)	9 (9.4%)	10 (10.5%)	0.7670
	1–3 times a month	52 (27.4%)	27 (28.4%)	25 (26.3%)	
	Once a week	61 (32.1%)	32 (33.7%)	29 (30.5%)	
	Several times a week	56 (29.5%)	26 (27.4%)	30 (31.6%)	
	Once a day	1 (0.5%)	0 (0.0%)	1 (1.1%)	
	Several times a day	1 (0.5%)	1 (1.1%)	0 (0.0%)	
White meat	Never	11 (5.8%)	6 (6.3%)	5 (5.3%)	0.7479
	1–3 times a month	25 (13.2%)	10 (10.5%)	15 (15.8%)	
	Once a week	47 (24.7%)	24 (25.3%)	23 (24.2%)	
	Several times a week	103 (54.2%)	54 (56.8%)	49 (51.5%)	
	Once a day	3 (1.6%)	1 (1.1%)	2 (2.1%)	
	Several times a day	1 (0.5%)	0 (0.0%)	1 (1.1%)	
Fish ^1^	Never	9 (4.7%)	6 (6.4%)	3 (3.2%)	0.3380
	1–3 times a month	82 (43.4%)	38 (40.4%)	44 (46.3%)	
	Once a week	79 (41.8%)	43 (45.7%)	36 (37.9%)	
	Several times a week	19 (10.1%)	7 (7.5%)	12 (12.6%)	
	Once a day	0 (0.0%)	0 (0.0%)	0 (0.0%)	
	Several times a day	0 (0.0%)	0 (0.0%)	0 (0.0%)	
Eggs	Never	0 (0.0%)	0 (0.0%)	0 (0.0%)	0.9063
	1–3 times a month	21 (11.0%)	10 (10.5%)	11 (11.6%)	
	Once a week	52 (27.4%)	26 (27.4%)	26 (27.4%)	
	Several times a week	109 (57.4%)	54 (56.8%)	55 (57.8%)	
	Once a day	8 (4.2%)	5 (5.3%)	3 (3.2%)	
	Several times a day	0 (0.0%)	0 (0.0%)	0 (0.0%)	
Legumes	Never	14 (7.4%)	6 (6.3%)	8 (8.4%)	0.5646
	1–3 times a month	112 (58.9%)	61 (64.2%)	51 (53.7%)	
	Once a week	38 (20.0%)	17 (17.9%)	21 (22.1%)	
	Several times a week	25 (13.2%)	11 (11.6%)	14 (14.7%)	
	Once a day	1 (0.5%)	0 (0.0%)	1 (1.1%)	
	Several times a day	0 (0.0%)	0 (0.0%)	0 (0.0%)	
Potatoes	Never	7 (3.7%)	4 (4.2%)	3 (3.2%)	0.9606
	1–3 times a month	35 (18.4%)	18 (19%)	17 (17.9%)	
	Once a week	53 (27.9%)	28 (29.4%)	25 (26.3%)	
	Several times a week	89 (46.8%)	42 (44.2%)	47 (49.4%)	
	Once a day	6 (3.2%)	3 (3.2%)	3 (3.2%)	
	Several times a day	0 (0.0%)	0 (0.0%)	0 (0.0%)	
Fruits	Never	0 (0.0%)	0 (0.0%)	0 (0.0%)	0.4145
	1–3 times a month	5 (2.6%)	4 (4.2%)	1 (1.1%)	
	Once a week	10 (5.3%)	4 (4.2%)	6 (6.3%)	
	Several times a week	55 (28.9%)	24 (25.3%)	31 (32.6%)	
	Once a day	59 (31.1%)	33 (34.7%)	26 (27.4%)	
	Several times a day	61 (32.1%)	30 (31.6%)	31 (32.6%)	
Vegetables	Never	0 (0.0%)	0 (0.0%)	0 (0.0%)	0.5405
	1–3 times a month	3 (1.6%)	1 (1.1%)	2 (2.1%)	
	Once a week	3 (1.6%)	2 (2.1%)	1 (1.1%)	
	Several times a week	55 (29.0%)	24 (25.2%)	31 (32.6%)	
	Once a day	57 (30.0%)	27 (28.4%)	30 (31.6%)	
	Several times a day	72 (37.8%)	41 (43.2%)	31 (32.6%)	
Sweets	Never	7 (3.7%)	3 (3.2%)	4 (4.2%)	0.7752
	1–3 times a month	31 (16.3%)	15 (15.8%)	16 (16.8%)	
	Once a week	22 (11.6%)	12 (12.6%)	10 (10.5%)	
	Several times a week	71 (37.4%)	36 (37.9%)	35 (36.9%)	
	Once a day	38 (20.0%)	16 (16.8%)	22 (23.2%)	
	Several times a day	21 (11.0%)	13 (13.7%)	8 (8.4%)	
Instant soups or ready-made soups	Never	148 (77.9%)	73 (76.8%)	75 (78.9%)	0.2668
	1–3 times a month	35 (18.4%)	16 (16.8%)	19 (20.0%)	
	Once a week	6 (3.2%)	5 (5.3%)	1 (1.1%)	
	Several times a week	1 (0.5%)	1 (1.1%)	0 (0.0%)	
	Once a day	0 (0.0%)	0 (0.0%)	0 (0.0%)	
	Several times a day	0 (0.0%)	0 (0.0%)	0 (0.0%)	
Canned meat	Never	142 (74.8%)	70 (73.7%)	72 (75.8%)	0.5489
	1–3 times a month	46 (24.2%)	24 (25.2%)	22 (23.1%)	
	Once a week	1 (0.5%)	1 (1.1%)	0 (0.0%)	
	Several times a week	1 (0.5%)	0 (0.0%)	1 (1.1%)	
	Once a day	0 (0.0%)	0 (0.0%)	0 (0.0%)	
	Several times a day	0 (0.0%)	0 (0.0%)	0 (0.0%)	
Canned vegetables	Never	12 (6.3%)	4 (4.2%)	8 (8.4%)	0.4694
	1–3 times a month	63 (33.1%)	33 (34.7%)	30 (31.5%)	
	Once a week	46 (24.2%)	24 (25.3%)	22 (23.2%)	
	Several times a week	63 (33.2%)	31 (32.6%)	32 (33.7%)	
	Once a day	4 (2.1%)	3 (3.2%)	1 (1.1%)	
	Several times a day	2 (1.1%)	0 (0.0%)	2 (2.1%)	
Fruit juices	Never	34 (17.9%)	17 (17.9%)	17 (17.9%)	0.8182
	1–3 times a month	74 (39.0%)	39 (41.0%)	35 (36.8%)	
	Once a week	29 (15.3%)	14 (14.7%)	15 (15.8%)	
	Several times a week	41 (21.6%)	21 (22.1%)	20 (21.1%)	
	Once a day	7 (3.7%)	3 (3.2%)	4 (4.2%)	
	Several times a day	5 (2.6%)	1 (1.1%)	4 (4.2%)	
Vegetable or fruit–vegetable juices	Never	56 (29.5%)	29 (30.5%)	27 (28.4%)	0.6967
	1–3 times a month	82 (43.2%)	37 (38.9%)	45 (47.4%)	
	Once a week	22 (11.5%)	14 (14.7%)	8 (8.4%)	
	Several times a week	22 (11.5%)	11 (11.6%)	11 (11.6%)	
	Once a day	5 (2.6%)	3 (3.2%)	2 (2.1%)	
	Several times a day	3 (1.7%)	1 (1.1%)	2 (2.1%)	
Hot sweetened drinks	Never	90 (47.4%)	44 (46.3%)	46 (48.4%)	0.6133
	1–3 times a month	11 (5.8%)	8 (8.4%)	3 (3.2%)	
	Once a week	1 (0.5%)	0 (0.0%)	1 (1.1%)	
	Several times a week	12 (6.3%)	6 (6.3%)	6 (6.3%)	
	Once a day	16 (8.4%)	7 (7.4%)	9 (9.4%)	
	Several times a day	60 (31.6%)	30 (31.6%)	30 (31.6%)	
Carbonated or non-carbonated sweetened beverages	Never	90 (47.4%)	47 (49.4%)	43 (45.2%)	0.7596
	1–3 times a month	82 (43.2%)	41 (43.2%)	41 (43.2%)	
	Once a week	13 (6.8%)	5 (5.3%)	8 (8.4%)	
	Several times a week	4 (2.1%)	2 (2.1%)	2 (2.1%)	
	Once a day	1 (0.5%)	0 (0.0%)	1 (1.1%)	
	Several times a day	0 (0.0%)	0 (0.0%)	0 (0.0%)	
Energy drinks	Never	177 (93.2%)	90 (94.7%)	87 (91.6%)	0.3887
	1–3 times a month	13 (6.8%)	5 (5.3%)	8 (8.4%)	
	Once a week	0 (0.0%)	0 (0.0%)	0 (0.0%)	
	Several times a week	0 (0.0%)	0 (0.0%)	0 (0.0%)	
	Once a day	0 (0.0%)	0 (0.0%)	0 (0.0%)	
	Several times a day	0 (0.0%)	0 (0.0%)	0 (0.0%)	
Water	Never	7 (3.7%)	5 (5.3%)	2 (2.1%)	0.8130
	1–3 times a month	5 (2.6%)	3 (3.2%)	2 (2.1%)	
	Once a week	4 (2.1%)	2 (2.1%)	2 (2.1%)	
	Several times a week	21 (11.1%)	11 (11.5%)	10 (10.5%)	
	Once a day	13 (6.8%)	5 (5.3%)	8 (8.4%)	
	Several times a day	140 (73.7%)	69 (72.6%)	71 (74.8%)	
Alcoholic drinks	Never	42 (22.1%)	17 (17.9%)	25 (26.3%)	0.3258
	1–3 times a month	72 (37.9%)	38 (39.9%)	34 (35.8%)	
	Once a week	43 (22.6%)	22 (23.2%)	21 (22.1%)	
	Several times a week	29 (15.3%)	15 (15.8%)	14 (14.7%)	
	Once a day	3 (1.6%)	3 (3.2%)	0 (0.0%)	
	Several times a day	1 (0.5%)	0 (0.0%)	1 (1.1%)	

^1^ NCF: *n* = 94; MCI—mild cognitive impairment; NCF—normal cognitive function.

## Data Availability

The data presented in this study are available on request from the corresponding author (J.W.).

## Supplementary Materials

The following supporting information can be downloaded at: https://www.mdpi.com/article/10.3390/nu16050644/s1, Table S1: STROBE statement checklist; Table S2: Characteristics of women and men with NCF and MCI; Table S3: Comparison of body composition between women and men with NCF and MCI; Table S4: Comparison of densitometric parameters between women and men with NCF and MCI; Table S5: Comparison of intake of energy and selected macro- and micronutrients between women and men with NCF and MCI; Table S6: Comparison of frequency of consumption of selected food products between women and men with NCF and MCI.
