# Supplementary material for: Comparison of Eating Habits, Body Composition and Densitometric Parameters between Subjects with Normal Cognitive Function and Mild Cognitive Impairment: An Observational Study
Source: Nutrients. 2024 Feb 25;16(5):644. doi: 10.3390/nu16050644 (PMC10934958; doi:10.3390/nu16050644)
Supplement: Supplementary file 1 [file nutrients-16-00644-s001.zip › nutrients-2847564-supplementary.pdf]

Table S1. STROBE Statement—Checklist of items, which should be included in reports of observational studies

	Item No.	Recommendation	Page No.	Relevant Text from Manuscript
**Title and Abstract**	1	(*a*) Indicate the study's design with a commonly used term in the title or the abstract	1	Comparison of eating habits, body composition and densitometric parameters between subjects with normal cognitive function and mild cognitive impairment: An observational study.
		(*b*) In the abstract, provide an informative and balanced summary of what was performed and what was found	1	The role of nutrition in the ageing process of the brain is pivotal. Therefore, the study aimed to compare eating habits, body composition and densitometric parameters between subjects with normal cognitive function (NCF) and mild cognitive impairment (MCI). A total of 95 subjects with NCF (74% of women) and 95 individuals with MCI (77% of women) aged 50–70 years were studied. Densitometric parameters were evaluated using dual-energy X-ray absorptiometry methods. Eating habits were assessed using the food frequency questionnaire and 3-day diary records, and advanced glycation end products (AGEs) intake was calculated. Significant differences between groups were detected in terms of the %fat in the right arm (NCF vs. MCI: 38.4 (30.4 – 46.8) vs. 43.5 (35.5 – 49.2)%, p=0.0407). Moreover, the MCI group had a significantly lower intake of calcium (p=0.0010), phosphor (p=0.0411), vitamins B2 (p=0.0138) and B12 (p=0.0024) compared to the NCF group, with both groups also differing in the frequency of butter (p=0.0191) and fermented milk beverages (p=0.0398) intake. Analysis restricted to women showed significant differences between groups in right arm %fat, VAT mass, calcium, vitamins B2, B12, butter and fermented milk products intake, while in men, differences were detected in the intake of calcium, iodine, vitamin B1, water and AGEs. In conclusion, subjects with NCF and MCI have comparable densitometric variables but differ significantly in some body composition parameters and the intake of some food groups and nutrients.
**Introduction**				
Background/rationale	2	Explain the scientific background and rationale for the investigation being reported	1–2	Mild cognitive impairment (MCI) is an intermediate condition between normal cognitive function (NCF) and dementia. Many different definitions and diagnostic criteria for diagnosing MCI have been described in the literature. Typically, it is

assumed that MCI is characterised by mild memory impairment, which does not, however, disturb the subject's normal functioning [1]. It is estimated that MCI affects up to 15% of the global population aged 50 and over [2]. These individuals with MCI are at increased risk of developing dementia, with an annual risk of dementia of 10–15% compared to 1–2% for similarly aged people with NCF. Early diagnosis and therapy of MCI may postpone or prevent the development of dementia [3].

Eating habits and nutrition status are important modifiable risk factors for the development of MCI and dementia, as both may play an essential role in the ageing process [4]. It has been suggested that higher consumption of vegetables and fruits [5], fish [6] and nuts [7] is associated with better cognitive functions and a lower risk of developing dementia and that meat consumption may increase the risk of cognitive impairment [8]. The role of nutrients such as fatty acids, vitamins and minerals in developing cognitive disorders has also been investigated, but the results so far have been inconclusive. Nevertheless, some studies suggested that antioxidants, unsaturated fatty acids or some B vitamins may be protective [9–12]. It has also been suggested that advanced glycation end products (AGEs) intake may affect cognitive function. AGEs are formed mainly during the Maillard reaction [13], and higher levels of AGEs in the brains of subjects with Alzheimer's disease contribute to amyloid plaque deposition [14]. Moreover, higher AGEs concentrations in blood [15] and urine [16] were associated with more significant cognitive decline. However, only a few studies have evaluated AGEs intake in subjects with cognitive decline, suggesting that higher intake may be associated with faster cognitive impairment [17].

Nutritional status may also determine the prevalence of cognitive impairment, with some findings suggesting that loss of free-fat mass may be linked to cognitive decline [18]. However, the association between body composition and overall cognitive function is controversial, as other studies reported no associations between body composition and cognitive dysfunction [19]. Moreover, a previous meta-analysis showed that obesity and a higher body mass index (BMI) may be associated with an increased risk of dementia [20]. Some data also suggested that bone mineral density (BMD) is reduced in cognitively impaired individuals [21,22], but this has not been confirmed in other studies [23]. Furthermore, the underlying mechanisms

				for the association between cognition and body composition and densitometric parameters are not yet fully understood.
Objectives	3	State specific objectives, including any pre-specified hypotheses	2	Therefore, the primary aim of this study was to compare eating habits and nutritional value of diet and AGEs intake between subjects with NCF and MCI. The secondary objective included the evaluation of body composition and densitometric variables in MCI and NCF individuals. Moreover, we also performed separate analyses for men and women.
**Methods**				
Study design	4	Present the key elements of study design early in the paper	2	This observational study was conducted according to the Strengthening the Reporting of Observational Studies in Epidemiology (STROBE) [24] and the Declaration of Helsinki [25].
Setting	5	Describe the setting, locations and relevant dates, including periods of recruitment, exposure, follow-up and data collection	2	Participants were recruited from the Greater Poland Voivodeship from July 2021 to August 2022 by a physician at the Department of Pediatric Gastroenterology and Metabolic Diseases, Poznan University of Medical Sciences.
Participants	6	(*a*) *Cohort study*—Give the eligibility criteria and the sources and methods of selection of participants. Describe the methods of follow-up<br>*Case–control study*—Give the eligibility criteria and the sources and methods of case ascertainment and control selection. Give the rationale for the choice of cases and controls<br>*Cross-sectional study*—Give the eligibility criteria and the sources and methods of selection of participants	2-3	The inclusion criteria were age 50–70, Montreal Cognitive Assessment (MOCA) scores of 19–26 points (MCI group) and 27–30 points (NCF group), residing within the community. The exclusion criteria were MOCA scores < 19 points, history of depression treatment and/or Hamilton Depression Rating scale (HAM-D) test scores > 13 points, usage of cognitive enhancement drugs or psychotropic medications, excessive alcohol consumption (> 15 units per week), substance abuse disorders, mental health conditions, Parkinson's disease, Alzheimer's disease, dementia, anaemia, diabetes ≥ 10 years, severe chronic kidney and liver diseases, a previous cancer diagnosis with chemotherapy or radiotherapy within the last five years, a history of stroke, seizures in the past two years, a head injury leading to loss of consciousness or immediate post-injury confusion, hypothyroidism with current abnormal levels of thyrotropic hormone, any other severe chronic illnesses preventing participation in the study, high levels of physical activity, presence of implanted pacemakers, neurostimulators or other metallic components, including prosthetic implants, blindness, deafness, communication challenges or any other disabilities, which may hinder participation in the study.

		(*b*) *Cohort study*—For matched studies, give the matching criteria and the number of exposed and unexposed groups<br>*Case–control study*—For matched studies, give the matching criteria and the number of controls per case	4	Therefore, the final NCF group consisted of 95 participants. The MCI group also included 95 subjects. Subjects in the MCI group were selected from among 198 people who took part in the randomised controlled trial [35]. The MCI group was matched with the NCF group in terms of age, sex and BMI.
Variables	7	Clearly define all outcomes, exposures, predictors, potential confounders and effect modifiers. Give diagnostic criteria, if applicable	3	During the recruitment visit, physicians completed the MOCA and HAM-D questionnaire with potential participants, collected medical information and measured body weight and height. Participants also received a food frequency questionnaire and a 3-day food diary to complete at home for submission at the next visit, during which body composition and densitometric parameters were determined. All measurements were performed at the Department of Pediatric Gastroenterology and Metabolic Diseases, Poznan University of Medical Sciences.
Data sources/ measurement	8*	For each variable of interest, give the sources of data and details of the methods of assessment (measurement). Describe the comparability of assessment methods if there is more than one group	3–4	The MOCA questionnaire was used to allocate study participants to the MCI and NCF groups and evaluate visuospatial and executive function, naming, memory, attention, language, abstraction, delayed recall and orientation. A qualified physician, appropriately trained and certified for MOCA administration and scoring, conducted the assessment. MOCA scores within the range of 27–30 points are indicative of NCF, while scores within the range of 19–26 points suggest MCI, and scores below 19 points typically lead to a diagnosis of dementia [26].<br>The HAM-D questionnaire was used to assess the occurrence of depressive symptoms, with scores of ≥ 23 indicative of very severe depression and the range of 18–22 signifying severe depression. Scores within the range of 14–18 indicate moderate depression, while the range of 8–13 indicates mild depression, and < 7 denotes an absence of depression [27,28].<br>2.6 Anthropometric parameters<br>Body weight was measured using an electronic scale with an altimeter (Radwag, WPT 100/200 OW, Radom, Poland) and was performed without shoes and in underwear with an accuracy of 0.1 kg. Body height was measured with an accuracy of 0.1 cm. BMI was calculated to assess the nutritional status of the study population

according to the World Health Organization (WHO) criteria. Malnutrition was defined as a BMI of ≤ 18.5 kg/m²; 18.5–24.9 kg/m² was considered within the normal weight range; overweight was classified as a BMI between 25 and 29.9 kg/m²; and obesity was represented by a BMI of ≥ 30 kg/m² [29].

2.7 Body composition

Body composition analysis was assessed with dual-energy X-ray absorptiometry (DEXA) methods using the Hologic Discovery analyser (Bedford, Massachusetts, USA). The measurement included determining the percentage of body fat (%BF) in the total body and individual areas, such as arms, legs, trunk, male (android) and female (gynoid). Moreover, the visceral adipose tissue (VAT) content was determined, and the proportion of android to visceral fat distribution, as well as the trunk/leg index and fat mass index (FMI), were also calculated. The appendicular lean mass index (ALMI) and lean mass index (LMI) were used to determine the muscle mass content [30].

2.8 Densitometric parameters

Bone mineral content (BMC) and BMD at the lumbar spine (L1–L4) were analysed by DEXA using the Hologic Discovery DXA system (Bedford, MA, USA). All assessments were performed based on the International Society for Clinical Densitometry guidelines [31], with participants in their underwear and without shoes. All metal elements were removed before measurement. The WHO criteria were used to assess bone health status, with a T-score > -1 indicative of normal bone health, ≤ -1 but > -2.5 indicating osteopenia and a T-score ≤ -2.5 suggesting osteoporosis [32].

2.9 Eating habits

A self-administrative version of the Dietary Habits and Nutrition Beliefs Questionnaire (KomPAN) was used to assess participants' dietary habits. In our study, part B of the KomPAN questionnaire related to food frequency consumption was administered [33], with nutritional habits also evaluated using 3-day diary records. A qualified dietician instructed participants on how to complete the questionnaire and checked both questionnaires. The intake of macro- (fats, proteins, carbohydrates) and selected micronutrients (including fatty acids, vitamins and minerals) was calculated using the Aliant software version no: 87 (Anmarsoft,

				Gdańsk, Poland) based on the 3-day diary dietary energy value of the diet. Moreover, AGEs intake was calculated using the Uribarri et al. [34] database, which comprises the most commonly consumed foods and widely employed culinary techniques in the USA. Consequently, not all food items available in Poland were included in the database; therefore, the AGEs content was estimated by referencing similar foods with similar nutrient and ingredient profiles. In instances where the AGEs content of a specific food prepared with a particular culinary method was unavailable, the AGEs content of a comparable food prepared using a similar culinary method was utilised.
Bias	9	Describe any efforts to address potential sources of bias	4	The MCI group was matched with the NCF group in terms of age, sex and BMI.
Study size	10	Explain how the study size was arrived at	4	The minimum sample size was calculated as 75 subjects per group using the G*Power 3.1 software (University of Kiel, Kiel, Germany) to obtain a power of 80% ($\alpha = 0.05$, $\beta = 0.2$). Considering a maximum dropout rate of 20%, each group should contain at least 90 subjects. This calculation was based on the estimated differences in AGEs intake between groups, as determined in our preliminary study (data not published). We assumed that the differences in AGEs intake between the groups would amount to 1500 kU, with a standard deviation of 3250 kU.
Quantitative variables	11	Explain how quantitative variables were handled in the analyses. If applicable, describe which groupings were chosen and why	3-4	A qualified physician, appropriately trained and certified for MOCA administration and scoring, conducted the assessment. MOCA scores within the range of 27–30 points are indicative of NCF, while scores within the range of 19–26 points suggest MCI, and scores below 19 points typically lead to a diagnosis of dementia [26].<br>The HAM-D questionnaire was used to assess the occurrence of depressive symptoms, with scores of ≥ 23 indicative of very severe depression and the range of 18–22 signifying severe depression. Scores within the range of 14–18 indicate moderate depression, while the range of 8–13 indicates mild depression, and < 7 denotes an absence of depression [27,28].<br>Malnutrition was defined as a BMI of ≤ 18.5 kg/m²; 18.5–24.9 kg/m² was considered within the normal weight range; overweight was classified as a BMI between 25 and 29.9 kg/m²; and obesity was represented by a BMI of ≥ 30 kg/m² [29].

				The WHO criteria were used to assess bone health status, with a T-score > -1 indicative of normal bone health, ≤ -1 but > -2.5 indicating osteopenia and a T-score ≤ -2.5 suggesting osteoporosis [32].
Statistical methods	12	(*a*) Describe all statistical methods, including those used to control for confounding factors	4	Statistics were performed in the Statistica 13.0 program (TIBCO Software Inc., Palo Alto, CA, USA), with a p-value < 0.05 considered statistically significant. The normality of the distribution of variables was verified by the Shapiro–Wilk test. Due to the lack of normal distribution for most of the analysed variables, the characteristics of the study population were presented as median and interquartile range (IQR) or in the form of frequencies and percentages. The Mann–Whitney test was used for comparisons with unpaired groups; the Chi2 test was used to evaluate categorical variables; and the Spearman index was used to assess the correlation between selected parameters.
		(*b*) Describe any methods used to examine subgroups and interactions		
		(*c*) Explain how missing data were addressed		
		(*d*) *Cohort study*—If applicable, explain how loss to follow-up was addressed<br>*Case–control study*—If applicable, explain how the matching of cases and controls was addressed<br>*Cross-sectional study*—If applicable, describe the analytical methods taking account of the sampling strategy		
		(*e*) Describe any sensitivity analyses		
**Results**				
Participants	13*	(a) Report the numbers of individuals at each stage of the study—e.g., numbers potentially eligible, examined for eligibility, confirmed eligible, included in the study, completing follow-up and analysed	4-5	Figure 1 depicts the work's flow. Over 1,000 individuals expressed their interest in study participation, of whom 969 subjects were invited to the recruitment visit, and 671 subjects did not meet the inclusion criteria, withdrew from the study or were excluded due to loss of contact. Ultimately, 99 people were included in the NCF group, but 4 individuals dropped out of the study. Therefore, the final NCF group consisted of 95 participants. The MCI group also included 95 subjects. Subjects in

		(b) Give reasons for non-participation at each stage		the MCI group were selected from among 198 people who took part in the randomised controlled trial [35].
		(c) Consider the use of a flow diagram		
Descriptive data	14*	(a) Give the characteristics of study participants (e.g., demographic, clinical, social) and information on the exposures and potential confounders	5	Table 1
		(b) Indicate the number of participants with missing data for each variable of interest	5–12	Tables 1–5; S2–S6
		(c) *Cohort study*—Summarise follow-up time (e.g., average and total amount)	-	N/A
Outcome data	15*	*Cohort study*—Report the numbers of outcome events or summary measures over time	-	N/A
		Case–control study—Report the numbers in each exposure category or summary measures of exposure	-	N/A
		Cross-sectional study—Report the numbers of outcome events or summary measures	-	N/A
Main results	16	(*a*) Give the unadjusted estimates and—if applicable—confounder-adjusted estimates and their precision (e.g., 95% confidence interval). Make clear which	5–12	Tables 1–5

		confounders were adjusted for and why they were included		
		(*b*) Report the category boundaries if continuous variables were categorised		
		(*c*) If relevant, consider translating the estimates of relative risk into absolute risk for a meaningful time period		
Other analyses	17	Report other analyses performed—e.g., analyses of subgroups and interactions and sensitivity analyses	Supplementary Material	Table S2–S6
**Discussion**				
Key results	18	Summarise the key results with reference to study objectives	12	There were no differences in densitometric parameters between subjects with NCF and MCI. However, some differences between groups in terms of body composition parameters and the nutritional value of diet and some food product intakes were noted.
Limitations	19	Discuss the limitations of the study, taking into account the sources of potential bias or imprecision Discuss both the direction and magnitude of any potential bias	15	The study's limitations include the allocation of study participants to the MCI and NCF groups based only on the MOCA test results. Another limitation is using the self-completed version of the KomPAN questionnaire and a 3-day food diary, which may have introduced reporting bias in food intake. However, a qualified dietitian instructed participants on completing the survey and verified whether the study participants had completed both questionnaires correctly. In addition, subjects with MCI may not be able to accurately assess their dietary intake using subjective methods. Indeed, our previous study showed that objective rather than subjective methods are more reliable in assessing physical activity in MCI individuals [41]. Another limitation is that the KomPAN questionnaire is validated only for individuals up to 65 years of age. However, the choice of this questionnaire resulted from the initial study inclusion criteria, which was 50–65 years of age, but due to difficulties in recruiting an adequate number of subjects with MCI, the age criteria

				were expanded to 50–70 years. In addition, dietary supplement intake was not monitored. Furthermore, it should be noted that the AGEs database utilised in the current study was originally established in the USA, and there are significantly diverse dietary patterns between the USA and Poland. This database exclusively includes carboxymethyl-lysine as an indicator of AGEs, omitting other significant markers, such as carboxyethyl-lysine and methylglyoxal-derived hydroimidazolone 1, and it has a limited number of records.
Interpretation	20	Give a cautious overall interpretation of the results, considering the objectives, limitations, multiplicity of analyses, results from similar studies and other relevant evidence	12–15	Previously, it has been suggested that cognitive decline may affect the bone remodelling process [36]. Indeed, Zhang et al. [21] showed a significant decrease in BMD in subjects with Alzheimer's disease compared to NCF participants and a positive correlation between BMD and scores obtained in the Minimal Mental State Examination (MMSE) scale. In addition, the receiver operator characteristic curve analysis indicated that this densitometric variable could be used to distinguish cognitive impairment participants from NCF individuals. Similar results were obtained by Lee et al. [22], who reported that cognitive impairment was associated with lower BMD at the lumbar spine and total hip. However, patients with Alzheimer's disease were compared to subjects with subjective cognitive impairment in this study. Additionally, Lin et al. [37] reported that BMD is effective in predicting MMSE scores. Furthermore, Noh et al. [38] showed that a higher BMC at the arm was associated with a decreased probability of MCI development, but this association was no longer significant after adjusting for potential confounding factors. In contrast, Patel et al. [23] observed no association between cognitive function and densitometric markers, which is in line with our results. No association between cognition and bone parameters was also found by Nourhashemi et al. [39]. We hypothesise that the differences between the studies' results may be due to differences in the age and sex of the study participants.<br>It is also speculated that changes in body composition might be associated with cognitive decline. To date, several studies reported that lower free-fat mass is related to a higher risk of developing MCI [18,39]. A decrease in lean mass is generally observed with ageing and is frequently associated with low diet quality [40] and low physical activity [41], both of which are also common in cognitive decline. Other mechanisms involved in this process may be associated with oxidative stress, the

inflammatory process and hormonal changes [39]. However, some studies suggested that an increase in %BF may be associated with better cognitive functions [42]. It is assumed that the higher concentrations of leptin observed in subjects with higher fat tissue content may be responsible for the protective effect of preventing cognitive disorders [43]. Moreover, a higher %BF is often related to a higher BMI, while BMI is positively correlated with white matter volume [44]. In contrast, another study demonstrated a lack of association between body composition and cognition, but due to the small sample size, the statistical power of this study was low [19]. Differences in the results in selected studies may be due to the use of different tools to assess cognitive function and measure body composition or differences in subjects' race or ethnicity. In our study, significant differences between groups were detected only for %BF in the right arm, with lower values found in the NCF group. Notably, a subgroup analysis confirmed these differences only in the women group. Moreover, women in the MCI group also had a significantly higher VAT mass than women in the NCF group. The underlying mechanism for the observed disparity in %BF in the right arm remains unclear. We hypothesise that this may be linked to the handedness of participants, although, due to the lack of data on their dominant hands, this remains speculative. The observed differences between the MCI and NCF groups in body composition parameters may also be associated with higher physical activity in subjects with NCF compared to MCI individuals. Indeed, our previous study showed that NCF participants, compared to people with MCI, are characterised by higher total and moderate physical activity and lower sedentary activity measured by the ActiGraph [41].

Some nutrients, such as antioxidants, B vitamins or unsaturated fatty acids, could potentially have significant impacts on brain function [45,46]. Therefore, it is suggested that the intake of some nutrients may play an important role in preventing cognitive disorders. To date, several studies have compared the eating habits of subjects with MCI with the eating habits of individuals with NCF, providing unequivocal results. In our study, significant differences between MCI and NCF groups were detected in the intake of calcium, phosphor, vitamin B2 and vitamin B12, with lower intake observed in MCI individuals. Differences between groups in the intake of calcium, vitamin B2 and vitamin B12 were confirmed in a separate

analysis for women. In addition, analysis restricted to men also showed significant differences between groups in the intake of calcium, iodine, vitamin B1 and water. Indeed, previous findings suggested that B vitamins might modulate the prevalence of cognitive decline. It is well known that vitamin B12 is involved in the DNA methylation process and the conversion of homocysteine to methionine, while higher levels of homocysteine may potentially result in a neurotoxic effect [47]. As higher concentrations of homocysteine were noted in subjects with dementia, it was speculated that homocysteine levels may predict the risk of development of cognitive decline [48]. In addition, higher vitamin B2 intake was noted in subjects with higher MMSE scores by Requejo et al. [49]. Moreover, Ozawa et al. [50] observed that higher self-reported intake of some minerals, such as potassium, calcium and magnesium, was associated with a lower risk of developing cognitive impairment. These findings are partly in line with our results, as we found a low intake of calcium and phosphorus in subjects with MCI. In contrast, Cherbuin et al. [51] demonstrated that higher potassium and iron consumption increased the risk of developing MCI. The mechanism through which the risk of cognitive decline changes with mineral intake is unclear, but it is suggested that, for potassium, this could be associated with an antihypertension effect [52]. Additionally, several studies reported the protective effects of dietary antioxidants on cognition [49,52], but we did not observe any differences in the intake of antioxidant vitamins between subjects with NCF and MCI. Similarly, no differences in fatty acid intake were observed between the groups, while previous results suggested that the intake of unsaturated fats, especially monounsaturated fatty acids and n-3 polyunsaturated fatty acids, might protect against cognitive decline [11]. In addition, there were no differences between groups in the present study regarding the intake of calories, fats, proteins and carbohydrates, while some previous studies suggested that diet macronutrient distribution might affect cognitive function [53]. In contrast, similar to our study, other studies did not demonstrate differences in energy or macronutrient intake between subjects with Alzheimer's disease, MCI and controls [54]. We speculate that potential differences between the studies' results may be related to different dietary assessment methods. Additionally, current intake may not reflect the intake, which has occurred over the past years.

Previously, higher AGEs levels were associated with greater cognitive decline through the effects on β-amyloid and tau protein metabolism [16]. Moreover, Fleitas et al. [55] postulated that AGEs may modify the precursor form of brain-derived neurotrophic factor, leading to neuronal apoptosis by inducing the processing of the p75 neurotrophic receptor. Therefore, we hypothesised that MCI and NCF subjects might differ significantly in AGEs intake, but our results did not confirm this hypothesis, as we noted no differences between the groups. However, a separate analysis for men showed that MCI subjects intake significantly higher amounts of AGEs than NCF individuals. Moreover, the calculated AGEs intake in the present study was similar to the results reported among healthy subjects [56]. Nevertheless, West et al. [17] showed that higher dietary AGEs intake was associated with faster cognitive decline. Moreover, Lotan et al. [57] found that a decrease in AGE intake improves cognitive function in subjects with diabetes. Due to unequivocal results, further studies are needed to assess whether subjects with MCI differ from subjects with NCF in AGEs consumption.

Previous studies suggested that healthy eating habits may protect against the development of cognitive impairment. However, Milte et al. [58] showed that diet variety, not quality, was associated with cognitive function. Nevertheless, a potential mechanism by which a healthy diet may protect against cognitive decline is associated with, among other things, a positive effect of diet on the cardiovascular system [59]. Therefore, we hypothesise that subjects with MCI may significantly differ from NCF participants in the frequency of intake of selected food products. However, our study comparing the intake of selected food groups found that MCI and NCF subjects differed only in the frequency of butter and fermented milk beverages intake, with more frequent consumption in the NCF group. However, when we conducted a separate analysis for each sex, these associations were detected only in women. Additionally, Wang et al. [60] demonstrated a higher intake of animal oil in the NCF elderly Chinese subjects compared to MCI participants. Nevertheless, these findings were somewhat surprising, despite a previous meta-analysis reporting that higher milk consumption was associated with a reduced risk of cognitive decline [61]. We rather expected to find significant differences between groups in the frequency of fruit and vegetable intake, as their higher consumption is

				associated with a lower incidence of cognitive disorders [62]. Okubo et al. [63] also showed that plant and fish food pattern was associated with higher scores obtained in the MOCA test. Moreover, higher adherence to the Mediterranean diet—which is characterised by high consumption of vegetables and fruits, legumes and cereals, moderate-to-high intake of fish and other sources of unsaturated fatty acids, low-to-moderate intake of dairy products, low intake of meat and saturated fatty acids, and a regular but moderate intake of alcohol—is a known protective factor against cognitive disorders [64]. We speculate that our study may have had inadequate power to detect significant differences in the intake of other food groups.<br>This study's strengths include strict and clearly defined inclusion and exclusion criteria and the use of propensity score matching to match both groups in terms of age, sex and BMI. Moreover, two methods were used (the KomPAN survey and a 3-day food diary) to determine the eating habits of the study population. Furthermore, this is one of the first studies comparing AGEs intake between subjects with NCF and MCI.<br>The study's limitations include the allocation of study participants to the MCI and NCF groups based only on the MOCA test results. Another limitation is using the self-completed version of the KomPAN questionnaire and a 3-day food diary, which may have introduced reporting bias in food intake. However, a qualified dietitian instructed participants on completing the survey and verified whether the study participants had completed both questionnaires correctly. In addition, subjects with MCI may not be able to accurately assess their dietary intake using subjective methods. Indeed, our previous study showed that objective rather than subjective methods are more reliable in assessing physical activity in MCI individuals [41].
Generalisability	21	Discuss the generalisability (external validity) of the study results	15	This study's strengths include strict and clearly defined inclusion and exclusion criteria and the use of propensity score matching to match both groups in terms of age, sex and BMI. Moreover, two methods were used (the KomPAN survey and a 3-day food diary) to determine the eating habits of the study population. Furthermore, this is one of the first studies comparing AGEs intake between subjects with NCF and MCI.
**Other information**				

Funding	22	Give the source of funding and the role of the funders for the present study and, if applicable, for the original study on which the present article is based	16	This research was funded by the National Science Center, grant number UMO-2017/27/B/NZ7/02924. The APC was funded by the Department of Pediatric Gastroenterology and Metabolic Diseases, Poznan University of Medical Sciences.

*Give the information separately for cases and controls in case–control studies and—if applicable—for exposed and unexposed groups in cohort and cross-sectional studies.

Note: An "Explanation and Elaboration" article discusses each checklist item and gives the methodological background and published examples of transparent reporting. The STROBE checklist is best used in conjunction with this article (freely available on the websites of *PLOS Medicine* at http://www.plosmedicine.org/, *Annals of Internal Medicine* at http://www.annals.org/ and *Epidemiology* at http://www.epidem.com/). Information on the STROBE initiative is available at www.strobe-statement.org.

Table S2. Characteristics of women and men with NCF and MCI.

	Median (IQR)					
	Women (n = 143)		p	Men (n = 47)		p
	NCF (n = 70)	MCI (n = 73)		NCF (n = 25)	MCI (n = 22)	
Age [years]	56 (53 – 61)	56 (52 – 62)	0.7198	57 (53 – 61)	58 (53 – 60)	0.6454
Weight [kg]	68.00 (60.00 – 80.00)	68.50 (61.00 – 78.50)	0.6893	94.20 (84.00 – 104.80)	87.15 (81.00 – 92.60)	0.1657
Height [m]	1.64 (1.61 – 1.68)	1.63 (1.58 – 1.67)	0.0622	1.78 (1.76 – 1.83)	1.76 (1.73 – 1.79)	0.1320
BMI [kg/m^2]	25.42 (22.24 – 29.03)	26.40 (23.60 – 29.76)	0.1499	28.24 (26.37 – 33.08)	27.80 (25.75 – 28.70)	0.4117
HAM-D [points]	4 (1 – 6)	3 (1 – 6)	0.5188	3 (0 – 6)	1 (0 – 3)	0.3282

BMI—body mass index; HAM-D—Hamilton Depression Rating scale; IQR—interquartile range; MCI—mild cognitive impairment; NCF—normal cognitive function.

Table S3. Comparison of body composition between women and men with NCF and MCI.

		Median (IQR)					
		Women (*n* = 143)		*p*	Men (*n* = 47)		*p*
		NCF (*n* = 70)	MCI (*n* = 73)		NCF (*n* = 25)	MCI (*n* = 22)	
BF [%]	Left arm	44.8 (36.5 – 50.4)	47.0 (43.1 – 51.1)	0.1581	27.3 (23.3 – 31.6)	26.8 (24.4 – 32.8)	0.5434
	Right arm	43.6 (34.8 – 49.2)	45.1 (42.3 – 49.8)	0.0456	28.2 (24.4 – 32.1)	27.8 (24.8 – 30.3)	0.8394
	Trunk	35.8 (29.5 – 40.4)	38.6 (33.0 – 42.2)	0.0783	31.3 (28.9 – 36.8)	31.3 (29.3 – 35.4)	0.8813
	Left leg	40.1 (36.1 – 44.6)	41.7 (38.4 – 45.3)	0.2173	24.2 (20.8 – 27.6)	25.5 (23.3 – 27.7)	0.3996
	Right leg	40.7 (36.0 – 44.9)	42.3 (38.4 – 44.7)	0.2773	24.3 (20.8 – 29.9)	24.8 (22.3 – 27.5)	0.7734
	Total	37.4 (32.4 – 42.5)	39.9 (35.6 –42.5)	0.0861	29.4 (25.5 – 32.7)	28.3 (25.9 – 32.1)	0.8813
	Male (android)	37.7 (29.3 – 42.7)	39.8 (34.2 – 44.0)	0.0730	35.5 (33.4 – 41.9)	36.4 (32.8 – 40.5)	1.0000
	Female (gynoid)	39.9 (36.4 – 43.5)	41.2 (38.9 –44.1)	0.1735	26.5 (23.8 – 29.0)	25.9 (23.8 – 29.4)	0.8729
FMI [kg/m^2]		9.0 (7.2 – 12.0)	10.4 (8.4 – 12.5)	0.0898	7.8 (6.5 – 10.5)	7.6 (6.9 – 8.4)	0.9066
Android/gynoid ratio		0.9 (0.8 – 1.0)	1.0 (0.9 – 1.0)	0.2614	1.3 (1.2 – 1.4)	1.4 (1.3 – 1.4)	0.3759
Trunk/leg fat ratio		1.0 (0.8 – 1.1)	1.0 (0.8 – 1.1)	0.5581	1.4 (1.3 – 1.7)	1.4 (1.3 – 1.6)	0.9915
VAT [g]		462.0 (304.0 –694.0)	588.0 (399.0 – 723.0)	0.0487	869.0 (672.0 – 1143.0)	759.5 (703.0 –1091.0)	0.9915
LMI [kg/m^2]		14.8 (13.9 – 16.1)	15.0 (14.0 – 16.2)	0.4961	18.9 (17.5 – 20.6)	18.3 (17.9 –19.0)	0.2449
ALMI [kg/m^2]		6.2 (5.7 – 6.9)	6.3 (5.8 – 6.8)	0.9436	8.6 (8.0 – 9.2)	8.2 (7.6 – 8.5)	0.0768

ALMI—appendicular lean mass index; BF—body fat; FMI—fat mass index; IQR—interquartile range; LMI—lean mass index; MCI—mild cognitive impairment; NCF—normal cognitive function; VAT—visceral adipose tissue.

Table S4. Comparison of densitometric parameters between women and men with NCF and MCI.

		Median (IQR)					
		**Women (*n* = 142)**		*p*	**Men (*n* = 47)**		*p*
		**NCF (*n* = 70)**	**MCI (*n* = 72)**		**NCF (*n* = 25)**	**MCI (*n* = 22)**	
Lumbar spine (L1–L4)	BMC [g]	58.12 (50.45 – 64.91)	57.19 (49.36 – 64.88)	0.9333	79.5 (64.6 – 84.5)	70.80 (63.44 – 82.99)	0.3320
	BMD [g/cm^2]	0.95 (0.85 – 1.07)	0.98 (0.88 – 1.07)	0.6937	1.08 (1.01 – 1.19)	1.06 (0.93 – 1.14)	0.3061
	Z-score	0.40 (-0.70 – 1.40)	0.60 (-0.25 – 1.40)	0.5484	0.30 (-0.20 – 1.50)	0.40 (-0.70 – 1.50)	0.6236
	T-score	-0.90 (-1.80 – 0.20)	-0.60 (-1.50 – 0.20)	0.6696	-0.10 (-0.80 – 0.90)	-0.30 (-1.40 – 0.40)	0.6236

BMC—bone mineral content; BMD—bone mineral density; IQR—interquartile range; MCI—mild cognitive impairment; NCF—normal cognitive function.

Table S5. Comparison of intake of energy and selected macro- and micronutrients between women and men with NCF and MCI.

	**Median (IQR)**					
	**Women (*n* = 143)**		*p*	**Men (*n* = 47)**		*p*
	**NCF (*n* = 70)**	**MCI (*n* = 73)**		**NCF (*n* = 25)**	**MCI (*n* = 22)**	
Energy [kcal]	1784 (1451 –2020)	1705 (1526 – 1925)	0.3964	2230 (1814 – 2752)	2161 (1701 – 3141)	0.9405
Protein [g]	69.1 (57.4 – 82.4)	66.2 (56.2 – 77.7)	0.2590	94.6 (76.5 – 113.4)	86.4 (73.7 – 115.1)	0.9151
Protein [%]	15.7 (13.9 – 16.9)	15.3 (14.0 – 17.3)	0.7727	16.6 (14.8 – 18.5)	16.0 (14.0 – 18.2)	0.6237
Fat [g]	69.9 (54.8 – 84.6)	65.8 (56.7 – 81.3)	0.9791	94.0 (72.6 – 114.1)	90.2 (71.6 – 118.8)	0.8229
Fat [%]	34.7 (29.6 – 39.5)	35.3 (31.2 – 41.0)	0.1846	37.7 (32.9 – 41.5)	36.7 (34.0 – 39.6)	0.6932
Carbohydrate [g]	218.1 (185.8 – 260.6)	207.6 (178.5 – 259.6)	0.3942	241.4 (197.0 – 314.6)	260.9 (184.0 – 349.1)	0.5648
Carbohydrate [%]	48.2 (43.7 – 52.2)	49.2 (43.1 – 52.1)	0.8876	43.8 (38.6 – 46.3)	43.8 (37.1 – 47.1)	0.9575
Digestible carbohydrate [g]	197.2 (168.9 – 238.7)	188.2 (156.9 – 234.2)	0.3235	221.3 (181.2 – 276.5)	232.3 (170.9 – 315.3)	0.5867
Fibre [g]	20.3 (15.9 – 26.4)	21.6 (17.8 – 25.0)	0.5433	22.1 (17.3 – 27.4)	26.4 (17.2 – 34.5)	0.4117
Sugar [g]	72.9 (57.6 – 98.4)	69.3 (53.4 – 86.6)	0.2659	70.7 (48.8 – 95.5)	75.0 (50.1 – 123.6)	0.6014
Sugar [%]	17.0 (13.9 – 20.4)	16.0 (12.7 – 19.4)	0.2693	13.1 (9.0 – 16.7)	13.0 (11.1 – 16.9)	0.6620
SFA [g]	27.2 (18.3 – 32.1)	24.1 (18.4 – 30.4)	0.5367	34.0 (27.4 – 44.0)	29.9 (24.3 – 38.7)	0.3215
SFA [%]	13.0 (11.0 – 14.9)	12.8 (10.5 – 15.8)	0.9437	15.0 (11.5 – 16.4)	12.4 (11.4 – 14.2)	0.0732
MUFA [g]	23.2 (17.6 – 30.9)	24.3 (19.2 – 31.3)	0.8211	35.3 (23.6 – 42.2)	30.5 (24.7 – 40.4)	0.5577

MUFA [%]	12.0 (10.1 – 14.2)	12.3 (10.5 – 15.1)	0.3050	13.8 (11.4 – 16.4)	13.2 (12.1 – 14.4)	0.3214
n-3 [g]	1.7 (1.3 – 2.4)	1.7 (1.2 – 2.7)	0.7865	2.3 (1.6 – 2.8)	2.2 (1.5 – 3.3)	0.5016
n-3 [%]	0.9 (0.7 – 1.1)	0.9 (0.7 – 1.2)	0.8776	0.9 (0.6 – 1.1)	0.9 (0.7 – 1.3)	0.5632
n-6 [g]	8.1 (5.6 – 10.4)	8.5 (5.5 – 10.7)	0.8748	10.9 (8.4 – 13.4)	12.6 (7.8 – 17.3)	0.3010
n-6 [%]	3.8 (3.0 – 5.2)	4.1 (3.2 – 5.3)	0.4671	3.9 (3.1 – 5.0)	4.6 (3.5 – 6.3)	0.1896
PUFA [g]	10.3 (7.6 – 13.1)	10.9 (7.2 – 13.8)	0.7147	12.8 (10.4 – 15.4)	15.3 (10.4 – 18.9)	0.2242
PUFA [%]	4.7 (3.9 – 7.0)	5.3 (4.1 – 7.0)	0.3333	4.8 (4.0 – 6.3)	5.6 (4.6 – 8.1)	0.1246
LA [g]	7.8 (5.6 – 10.3)	8.4 (5.4 – 10.5)	0.8844	10.8 (8.3 – 13.2)	12.4 (7.5 – 16.2)	0.3214
LA [%]	3.8 (3.0 – 5.0)	4.1 (3.1 – 5.2)	0.4770	3.8 (3.1 – 4.9)	4.6 (3.5 – 6.3)	0.2200
ALA [g]	1.6 (1.1 – 2.1)	1.5 (1.1 – 2.1)	0.5645	1.7 (1.2 – 2.3)	2.0 (1.4 – 3.1)	0.4297
ALA [%]	0.8 (0.6 – 1.0)	0.8 (0.6 – 1.0)	0.8855	0.8 (0.5 – 1.0)	0.9 (0.6 – 1.0)	0.4645
DHA [g]	0.1 (0.0 – 0.1)	0.1 (0.0 – 0.1)	0.4510	0.1 (0.0 – 0.1)	0.1 (0.0 – 0.1)	0.8008
EPA [g]	0.0 (0.0 – 0.0)	0.0 (0.0 – 0.1)	0.1182	0.0 (0.0 – 0.0)	0.0 (0.0 – 0.1)	0.7804
Cholesterol [mg]	312.0 (206.6 – 357.4)	273.9 (210.3 – 375.2)	0.8924	402.2 (281.1 – 500.9)	376.7 (286.3 – 452.6)	0.5867
Salt [g]	4.4 (3.5 – 6.0)	4.7 (3.5 – 5.9)	0.8494	6.9 (5.4 – 7.9)	6.4 (4.2 – 7.6)	0.3212
Sodium [mg]	1698.1 (1356.1 – 2338.7)	1825.3 (1348.6 – 2302.2)	0.8353	2684.6 (2078.0 – 3066.1)	2467.8 (1635.5 – 2953.9)	0.3112
Potassium [mg]	3071.0 (2480.5 – 3508.3)	2833.4 (2536.3 – 3440.9)	0.3776	3146.7 (2706.2 – 3872.5)	3646.2 (3054.3 – 4421.8)	0.3011

Calcium [mg]	705.7 (545.6 – 853.9)	584.4 (444.7 – 758.3)	0.0195	744.7 (647.7 – 876.5)	511.8 (434.7 – 729.4)	0.0165
Phosphor [mg]	1104.6 (979.5 – 1331.5)	1035.6 (884.5 – 1263.4)	0.0624	1335.9 (1188.1 – 1619.9)	1385.8 (1056.6 – 1707.4)	0.8229
Magnesium [mg]	281.8 (236.3 – 341.9)	278.8 (234.5 – 332.9)	0.7681	317.4 (275.7 – 361.3)	384.4 (284.8 – 454.3)	0.0942
Iron [mg]	10.4 (9.3 – 13.1)	9.8 (8.4 – 12.6)	0.3874	12.5 (11.1 – 15.9)	13.9 (8.9 – 16.6)	0.7816
Zinc [mg]	9.0 (7.5 – 10.6)	8.6 (7.0 – 10.1)	0.2615	11.4 (9.6 – 12.8)	11.5 (7.4 – 13.2)	0.8982
Copper [mg]	1.2 (1.0 – 1.5)	1.2 (1.0 – 1.5)	0.6512	1.2 (1.1 – 1.6)	1.6 (1.0 – 1.8)	0.2568
Manganese [mg]	4.3 (3.2 – 5.8)	4.2 (2.9 – 5.4)	0.5446	4.6 (3.0 – 5.8)	4.7 (2.7 – 7.1)	0.7169
Selenium [µg]	5.2 (3.0 – 11.2)	4.9 (2.3 – 14.7)	0.5828	13.4 (4.9 – 17.9)	3.6 (1.5 – 17.6)	0.1862
Iodine [µg]	33.0 (23.2 – 49.2)	37.0 (25.4 – 52.4)	0.7528	40.2 (33.6 – 64.8)	27.8 (15.5 – 60.0)	0.0486
Vit. A [µg]	979.2 (709.2 – 1414.8)	964.3 (719.1 – 1313.0)	0.7543	874.3 (728.0 – 1271.0)	1001.4 (597.2 – 1434.0)	0.9236
Retinol [µg]	333.9 (241.2 – 476.8)	333.8 (200.4 – 468.9)	0.4698	492.5 (352.9 – 747.5)	392.9 (242.0 – 491.8)	0.1274
β-carotene [µg]	3446.1 (1818.6 – 5320.8)	3153.4 (2078.9 – 5228.0)	0.9276	2130.8 (1328.2 – 3040.2)	2784.5 (1632.5 – 3858.4)	0.1222
Vit. D [µg]	1.8 (1.4 – 2.2)	2.1 (1.5 – 3.0)	0.0987	3.3 (1.8 – 4.3)	2.5 (1.9 – 4.7)	0.6465
Vit. E [mg]	9.9 (8.0 – 12.1)	10.5 (8.4 – 13.3)	0.4274	11.5 (7.4 – 14.0)	10.9 (7.7 – 15.8)	0.9745
Vit. K [µg]	15.1 (4.6 – 57.3)	13.6 (4.3 – 34.8)	0.5527	7.3 (3.3 – 35.1)	7.2 (4.0 – 17.4)	0.9830
Vit. B1 [mg]	1.1 (0.9 – 1.3)	1.0 (0.9 – 1.4)	0.8390	1.3 (1.1 – 1.5)	1.6 (1.3 – 2.0)	0.0180
Vit. B2 [mg]	1.7 (1.4 – 2.1)	1.6 (1.3 – 1.9)	0.0495	1.9 (1.7 – 2.4)	1.7 (1.4 – 2.5)	0.3041

Vit. B3 [mg]	13.6 (10.9 – 17.9)	13.9 (10.6 – 17.5)	0.7728	18.7 (15.7 – 24.2)	23.6 (17.8 – 26.2)	0.1471
Vit. B6 [mg]	1.6 (1.3 – 2.0)	1.6 (1.3 –1.9)	0.9516	1.8 (1.4 – 2.2)	2.1 (1.6 – 2.5)	0.1461
Folates [μg]	330.8 (263.8 – 388.2)	297.3 (268.0 – 361.2)	0.2919	336.9 (273.7 – 392.0)	362.2 (282.2 – 450.2)	0.5434
Vit. B12 [μg]	2.9 (2.4 – 3.8)	2.4 (1.8 – 3.5)	0.0159	4.6 (2.8 – 7.0)	3.0 (2.1 – 5.6)	0.1005
Vit. C [mg]	151.9 (100.4 – 206.3)	142.4 (97.2 – 192.1)	0.3375	122.0 (88.7 – 157.6)	124.8 (78.0 – 196.1)	0.9575
Water [g]	2045.1 (1554.0 – 2484.0)	2022.9 (1530.3 – 2522.6)	0.8353	2379.3 (1786.3 – 2782.1)	1950.8 (1399.9 – 2581.7)	0.0024
AGEs [kU]	9090.1 (6085.7 – 13,287.4)	8487.8 (6685.5 – 12,693.8)	0.7451	10,858.5 (8735.0 – 13,680.5)	14,726.2 (10,927.3 – 20,824.2)	0.0244

AGEs—advanced glycation end products; ALA—α-linolenic acid; DHA—docosahexaenoic acid; EPA—eicosapentaenoic acid; IQR—interquartile range; LA—linoleic acid; MCI—mild cognitive impairment; MUFA—monounsaturated fatty acids; NCF—normal cognitive function; PUFA—polyunsaturated fatty acids; SFA—saturated fatty acids; vit.—vitamin.

Table S6. Comparison of frequency of consumption of selected food products between women and men with NCF and MCI.

		n (%)					
		**Women (*n* = 143)**		*p*	**Men (*n* = 47)**		*p*
		**NCF (*n* = 70)**	**MCI (*n* = 73)**		**NCF (*n* = 25)**	**MCI (*n* = 22)**	
White bread	Never	3 (4.3%)	4 (5.5%)	0.8657	1 (4.0%)	0 (0.0%)	0.5363
	1–3 times a month	10 (14.3%)	15 (20.5%)		3 (12.0%)	5 (22.7%)	
	Once a week	8 (11.4%)	9 (12.3%)		1 (4.0%)	2 (9.1%)	
	Several times a week	17 (24.3%)	15 (20.6%)		8 (32.0%)	4 (18.2%)	
	Once a day	13 (18.6%)	15 (20.5%)		3 (12.0%)	5 (22.7%)	
	Several times a day	19 (27.1%)	15 (20.6%)		9 (36.0%)	6 (27.3%)	
Wholemeal bread	Never	3 (4.3%)	8 (11.0%)	0.6169	4 (16.0%)	2 (9.1%)	0.6295
	1–3 times a month	16 (22.9%)	13 (17.8%)		4 (16.0%)	4 (18.2%)	
	Once a week	5 (7.1%)	7 (9.6%)		3 (12.0%)	5 (22.7%)	
	Several times a week	29 (41.4%)	25 (34.2%)		11 (44.0%)	6 (27.3%)	
	Once a day	10 (14.3%)	13 (17.8%)		1 (4.0%)	3 (13.6%)	
	Several times a day	7 (10.0%)	7 (9.6%)		2 (8.0%)	2 (9.1%)	
White rice, pasta or small grains	Never	1 (1.4%)	3 (4.1%)	0.7836	0 (0.0%)	2 (9.1%)	0.3891
	1–3 times a month	26 (37.2%)	25 (34.2%)		7 (28.0%)	7 (31.8%)	
	Once a week	19 (27.1%)	24 (32.9%)		4 (16.0%)	5 (22.7%)	
	Several times a week	24 (34.3%)	21 (28.8%)		13 (52.0%)	8 (36.4%)	
	Once a day	0 (0.0%)	0 (0.0%)		1 (4.0%)	0 (0.0%)	
	Several times a day	0 (0.0%)	0 (0.0%)		0 (0.0%)	0 (0.0%)	
Buckwheat, oatmeal, whole-wheat pasta or other coarse-grain cereals	Never	4 (5.7%)	9 (12.3%)	0.6659	3 (12.0%)	3 (13.6%)	0.9369
	1–3 times a month	27 (38.5%)	23 (31.6%)		9 (36.0%)	9 (40.9%)	
	Once a week	9 (12.9%)	10 (13.7%)		5 (20.0%)	5 (22.7%)	
	Several times a week	20 (28.6%)	22 (30.1%)		6 (24.0%)	3 (13.6%)	
	Once a day	10 (14.3%)	9 (12.3%)		2 (8.0%)	2 (9.1%)	
	Several times a day	0 (0.0%)	0 (0.0%)		0 (0.0%)	0 (0.0%)	
Fast foods	Never	19 (27.1%)	24 (32.9%)	0.6401	5 (20.0%)	3 (13.6%)	0.6420
	1–3 times a month	50 (71.5%)	46 (63.0%)		18 (72.0%)	17 (77.2%)	

	Once a week	1 (1.4%)	3 (4.1%)		2 (8.0%)	1 (4.6%)	
	Several times a week	0 (0.0%)	0 (0.0%)		0 (0.0%)	1 (4.6%)	
	Once a day	0 (0.0%)	0 (0.0%)		0 (0.0%)	0 (0.0%)	
	Several times a day	0 (0.0%)	0 (0.0%)		0 (0.0%)	0 (0.0%)	
Fried foods	Never	0 (0.0%)	6 (8.2%)	0.1468	0 (0.0%)	1 (4.6%)	0.2727
	1–3 times a month	23 (32.8%)	25 (34.3%)		5 (20.0%)	5 (22.7%)	
	Once a week	18 (25.7%)	19 (26.0%)		8 (32.0%)	3 (13.6%)	
	Several times a week	27 (38.6%)	21 (28.8%)		12 (48.0%)	11 (50.0%)	
	Once a day	2 (2.9%)	2 (2.7%)		0 (0.0%)	2 (9.1%)	
	Several times a day	0 (0.0%)	0 (0.0%)		0 (0.0%)	0 (0.0%)	
Butter	Never	2 (2.8%)	9 (12.3%)	0.0173	2 (8.0%)	2 (9.1%)	0.8261
	1–3 times a month	9 (12.9%)	8 (11.0%)		4 (16.0%)	3 (13.6%)	
	Once a week	4 (5.7%)	9 (12.3%)		1 (4.0%)	3 (13.6%)	
	Several times a week	13 (18.6%)	14 (19.2%)		4 (16.0%)	5 (22.7%)	
	Once a day	17 (24.3%)	23 (31.5%)		6 (24.0%)	4 (18.3%)	
	Several times a day	25 (35.7%)	10 (13.7%)		8 (32.0%)	5 (22.7%)	
Lard[1]	Never	48 (68.6%)	50 (68.4%)	0.7203	17 (70.8%)	13 (59.0%)	0.6534
	1–3 times a month	20 (28.6%)	18 (24.7%)		7 (29.2%)	7 (31.8%)	
	Once a week	1 (1.4%)	3 (4.1%)		0 (0.0%)	1 (4.6%)	
	Several times a week	1 (1.4%)	1 (1.4%)		0 (0.0%)	1 (4.6%)	
	Once a day	0 (0.0%)	1 (1.4%)		0 (0.0%)	0 (0.0%)	
	Several times a day	0 (0.0%)	0 (0.0%)		0 (0.0%)	0 (0.0%)	
Oils or margarines[2]	Never	12 (17.4%)	23 (31.5%)	0.1187	6 (24.0%)	4 (18.2%)	0.4288
	1–3 times a month	8 (11.6%)	8 (10.9%)		4 (16.0%)	4 (18.2%)	
	Once a week	8 (11.6%)	7 (9.6%)		1 (4.0%)	3 (13.6%)	
	Several times a week	30 (43.5%)	17 (23.3%)		10 (40.0%)	5 (22.7%)	
	Once a day	9 (13.0%)	14 (19.2%)		3 (12.0%)	2 (9.1%)	
	Several times a day	2 (2.9%)	4 (5.5%)		1 (4.0%)	4 (18.2%)	
Milk	Never	7 (10.0%)	17 (23.3%)	0.4326	9 (36.0%)	3 (13.6%)	0.3086
	1–3 times a month	11 (15.7%)	12 (16.4%)		3 (12.0%)	4 (18.3%)	
	Once a week	6 (8.6%)	5 (6.9%)		0 (0.0%)	2 (9.1%)	
	Several times a week	11 (15.7%)	10 (13.7%)		5 (20.0%)	3 (13.6%)	
	Once a day	20 (28.6%)	17 (23.3%)		5 (20.0%)	5 (22.7%)	
	Several times a day	15 (21.4%)	12 (16.4%)		3 (12.0%)	5 (22.7%)	

Fermented milk beverages	Never	0 (0.0%)	2 (2.5%)	0.0235	1 (4.0%)	2 (9.1%)	0.9697
	1–3 times a month	7 (10.0%)	11 (15.1%)		8 (32.0%)	6 (27.3%)	
	Once a week	19 (27.1%)	9 (12.3%)		2 (8.0%)	1 (4.5%)	
	Several times a week	30 (42.9%)	45 (61.6%)		10 (40.0%)	10 (45.5%)	
	Once a day	13 (18.6%)	6 (8.2%)		4 (16.0%)	3 (13.6%)	
	Several times a day	1 (1.4%)	0 (0.0%)		0 (0.0%)	0 (0.0%)	
Quark	Never	1 (1.4%)	3 (4.1%)	0.4864	1 (4.0%)	3 (13.6%)	0.6082
	1–3 times a month	7 (10.0%)	15 (20.6%)		5 (20.0%)	5 (22.7%)	
	Once a week	18 (25.7%)	16 (21.9%)		5 (20.0%)	4 (18.2%)	
	Several times a week	30 (42.9%)	26 (35.6%)		8 (32.0%)	8 (36.3%)	
	Once a day	11 (15.7%)	11 (15.1%)		5 (20.0%)	1 (4.6%)	
	Several times a day	3 (4.3%)	2 (2.7%)		1 (4.0%)	1 (4.6%)	
Cheese	Never	2 (2.9%)	2 (2.7%)	0.5265	2 (8.0%)	0 (0.0%)	0.3086
	1–3 times a month	11 (15.7%)	16 (21.9%)		2 (8.0%)	6 (27.3%)	
	Once a week	20 (28.6%)	20 (27.4%)		4 (16.0%)	3 (13.6%)	
	Several times a week	25 (35.7%)	29 (39.7%)		11 (44.0%)	11 (50.0%)	
	Once a day	7 (10.0%)	5 (6.9%)		6 (24.0%)	2 (9.1%)	
	Several times a day	5 (7.1%)	1 (1.4%)		0 (0.0%)	0 (0.0%)	
Meats or sausages	Never	4 (5.7%)	5 (6.9%)	0.6620	2 (8.0%)	0 (0.0%)	0.6250
	1–3 times a month	9 (12.9%)	12 (16.3%)		2 (8.0%)	1 (4.6%)	
	Once a week	9 (12.9%)	14 (19.2%)		1 (4.0%)	1 (4.6%)	
	Several times a week	34 (48.5%)	33 (45.2%)		12 (48.0%)	15 (68.1%)	
	Once a day	10 (14.3%)	5 (6.9%)		6 (24.0%)	3 (13.6%)	
	Several times a day	4 (5.7%)	4 (5.5%)		2 (8.0%)	2 (9.1%)	
Red meat	Never	8 (11.4%)	10 (13.7%)	0.8425	1 (4.0%)	0 (0.0%)	0.6250
	1–3 times a month	20 (28.6%)	22 (30.2%)		7 (28.0%)	3 (13.6%)	
	Once a week	25 (35.7%)	21 (28.7%)		7 (28.0%)	8 (36.3%)	
	Several times a week	16 (22.9%)	20 (27.4%)		10 (40.0%)	10 (45.5%)	
	Once a day	0 (0.0%)	0 (0.0%)		0 (0.0%)	1 (4.6%)	
	Several times a day	1 (1.4%)	0 (0.0%)		0 (0.0%)	0 (0.0%)	
White meat	Never	5 (7.1%)	5 (6.9%)	0.4395	1 (4.0%)	0 (0.0%)	0.3540
	1–3 times a month	6 (8.6%)	14 (19.2%)		4 (16.0%)	1 (4.6%)	
	Once a week	16 (22.9%)	19 (26.0%)		8 (32.0%)	4 (18.2%)	
	Several times a week	42 (60.0%)	33 (45.2%)		12 (48.0%)	16 (72.6%)	

	Once a day	1 (1.4%)	2 (2.7%)		0 (0.0%)	0 (0.0%)	
	Several times a day	0 (0.0%)	0 (0.0%)		0 (0.0%)	1 (4.6%)	
Fish[2]	Never	5 (7.2%)	2 (2.7%)	0.4727	1 (4.0%)	1 (4.5%)	0.6153
	1–3 times a month	28 (40.6%)	34 (46.6%)		10 (40.0%)	10 (45.5%)	
	Once a week	31 (44.9%)	29 (39.7%)		12 (48.0%)	7 (31.8%)	
	Several times a week	5 (7.3%)	8 (11.0%)		2 (8.0%)	4 (18.2%)	
	Once a day	0 (0.0%)	0 (0.0%)		0 (0.0%)	0 (0.0%)	
	Several times a day	0 (0.0%)	0 (0.0%)		0 (0.0%)	0 (0.0%)	
Eggs	Never	0 (0.0%)	0 (0.0%)	0.5858	0 (0.0%)	0 (0.0%)	0.6115
	1–3 times a month	6 (8.6%)	9 (12.3%)		4 (16.0%)	2 (9.1%)	
	Once a week	19 (27.1%)	21 (28.8%)		7 (28.0%)	5 (22.7%)	
	Several times a week	40 (57.2%)	41 (56.2%)		14 (56.0%)	14 (63.7%)	
	Once a day	5 (7.1%)	2 (2.7%)		0 (0.0%)	1 (4.5%)	
	Several times a day	0 (0.0%)	0 (0.0%)		0 (0.0%)	0 (0.0%)	
Legumes	Never	2 (2.9%)	6 (8.2%)	0.3447	4 (16.0%)	2 (9.1%)	0.9323
	1–3 times a month	48 (68.6%)	40 (54.7%)		13 (52.0%)	11 (50.0%)	
	Once a week	11 (15.7%)	15 (20.6%)		6 (24.0%)	6 (27.3%)	
	Several times a week	9 (12.8%)	11 (15.1%)		2 (8.0%)	3 (13.6%)	
	Once a day	0 (0.0%)	1 (1.4%)		0 (0.0%)	0 (0.0%)	
	Several times a day	0 (0.0%)	0 (0.0%)		0 (0.0%)	0 (0.0%)	
Potatoes	Never	2 (2.9%)	2 (2.7%)	0.8455	2 (8.0%)	1 (4.5%)	0.3981
	1–3 times a month	12 (17.1%)	16 (21.9%)		6 (24.0%)	1 (4.5%)	
	Once a week	22 (31.4%)	17 (23.3%)		6 (24.0%)	8 (36.4%)	
	Several times a week	31 (44.3%)	35 (48.0%)		11 (44.0%)	12 (54.6%)	
	Once a day	3 (4.3%)	3 (4.1%)		0 (0.0%)	0 (0.0%)	
	Several times a day	0 (0.0%)	0 (0.0%)		0 (0.0%)	0 (0.0%)	
Fruits	Never	0 (0.0%)	0 (0.0%)	0.4057	0 (0.0%)	0 (0.0%)	0.0504
	1–3 times a month	0 (0.0%)	1 (1.4%)		4 (16.0%)	0 (0.0%)	
	Once a week	2 (2.9%)	3 (4.1%)		2 (8.0%)	3 (13.6%)	
	Several times a week	14 (20.0%)	23 (31.5%)		10 (40.0%)	8 (36.4%)	
	Once a day	26 (37.1%)	23 (31.5%)		7 (28.0%)	3 (13.6%)	
	Several times a day	28 (40.0%)	23 (31.5%)		2 (8.0%)	8 (36.4%)	
Vegetables	Never	0 (0.0%)	0 (0.0%)	0.4519	0 (0.0%)	0 (0.0%)	0.5574
	1–3 times a month	0 (0.0%)	1 (1.4%)		1 (4.0%)	1 (4.5%)	

	Once a week	0 (0.0%)	1 (1.4%)		2 (8.0%)	0 (0.0%)	
	Several times a week	14 (20.0%)	19 (26.0%)		10 (40.0%)	12 (54.6%)	
	Once a day	21 (30.0%)	24 (32.9%)		6 (24.0%)	6 (27.3%)	
	Several times a day	35 (50.0%)	28 (38.3%)		6 (24.0%)	3 (13.6%)	
Sweets	Never	1 (1.4%)	3 (4.1%)	0.6553	2 (8.0%)	1 (4.5%)	0.8225
	1–3 times a month	8 (11.4%)	12 (16.4%)		7 (28.0%)	4 (18.2%)	
	Once a week	10 (14.3%)	9 (12.3%)		2 (8.0%)	1 (4.5%)	
	Several times a week	27 (38.6%)	25 (34.3%)		9 (36.0%)	10 (45.5%)	
	Once a day	13 (18.6%)	17 (23.3%)		3 (12.0%)	5 (22.8%)	
	Several times a day	11 (15.7%)	7 (9.6%)		2 (8.0%)	1 (4.5%)	
Instant soups or ready-made soups	Never	55 (78.6%)	61 (83.6%)	0.6537	18 (72.0%)	14 (63.6%)	0.1984
	1–3 times a month	12 (17.1%)	11 1 (15.0%)		4 (16.0%)	8 (36.4%)	
	Once a week	2 (2.9%)	1 (1.4%)		3 (12.0%)	0 (0.0%)	
	Several times a week	1 (1.4%)	0 (0.0%)		0 (0.0%)	0 (0.0%)	
	Once a day	0 (0.0%)	0 (0.0%)		0 (0.0%)	0 (0.0%)	
	Several times a day	0 (0.0%)	0 (0.0%)		0 (0.0%)	0 (0.0%)	
Canned meat	Never	56 (80.0%)	60 (80.2%)	0.9903	14 (56.0%)	12 (54.6%)	0.5676
	1–3 times a month	14 (20.0%)	13 (17.8%)		10 (40.0%)	9 (40.9%)	
	Once a week	0 (0.0%)	0 (0.0%)		1 (4.0%)	0 (0.0%)	
	Several times a week	0 (0.0%)	0 (0.0%)		0 (0.0%)	1 (4.5%)	
	Once a day	0 (0.0%)	0 (0.0%)		0 (0.0%)	0 (0.0%)	
	Several times a day	0 (0.0%)	0 (0.0%)		0 (0.0%)	0 (0.0%)	
Canned vegetables	Never	3 (4.3%)	7 (9.6%)	0.3467	1 (4.0%)	1 (4.5%)	0.6615
	1–3 times a month	25 (35.7%)	24 (32.9%)		8 (32.0%)	6 (27.3%)	
	Once a week	18 (25.7%)	12 (16.4%)		6 (24.0%)	10 (45.4%)	
	Several times a week	22 (31.4%)	27 (37.0%)		9 (36.0%)	5 (22.8%)	
	Once a day	2 (2.9%)	1 (1.4%)		1 (4.0%)	0 (0.0%)	
	Several times a day	0 (0.0%)	2 (2.7%)		0 (0.0%)	0 (0.0%)	
Fruit juices	Never	9 (12.8%)	16 (21.9%)	0.6321	8 (32.0%)	1 (4.5%)	0.0929
	1–3 times a month	31 (44.3%)	28 (38.4%)		8 (32.0%)	7 (31.8%)	
	Once a week	13 (18.6%)	10 (13.7%)		1 (4.0%)	5 (22.8%)	
	Several times a week	14 (20.0%)	14 (19.2%)		7 (28.0%)	6 (27.3%)	
	Once a day	2 (2.9%)	2 (2.7%)		1 (4.0%)	2 (9.1%)	
	Several times a day	1 (1.4%)	3 (4.1%)		0 (0.0%)	1 (4.5%)	

Vegetable or fruit–vegetable juices	Never	20 (28.6%)	21 (28.8%)	0.2131	9 (36.0%)	6 (27.3%)	0.4228
	1–3 times a month	27 (38.6%)	33 (45.2%)		10 (40.0%)	12 (54.6%)	
	Once a week	12 (17.1%)	7 (9.6%)		2 (8.0%)	1 (4.5%)	
	Several times a week	8 (11.4%)	10 (13.7%)		3 (12.0%)	1 (4.5%)	
	Once a day	3 (4.3%)	0 (0.0%)		0 (0.0%)	2 (9.1%)	
	Several times a day	0 (0.0%)	2 (2.7%)		1 (4.0%)	0 (0.0%)	
Hot sweetened drinks	Never	32 (45.8%)	39 (53.4%)	0.7385	12 (48.0%)	7 (31.8%)	0.5293
	1–3 times a month	4 (5.7%)	2 (2.7%)		4 (16.0%)	1 (4.5%)	
	Once a week	0 (0.0%)	1 (1.4%)		0 (0.0%)	0 (0.0%)	
	Several times a week	5 (7.1%)	5 (6.9%)		1 (4.0%)	1 (4.5%)	0.5293
	Once a day	5 (7.1%)	6 (8.2%)		2 (8.0%)	3 (13.7%)	
	Several times a day	24 (34.3%)	20 (27.4%)		6 (24.0%)	10 (45.5%)	
Carbonated or non-carbonated sweetened beverages	Never	37 (52.9%)	37 (50.7%)	0.8550	10 (40.0%)	6 (27.3%)	0.6472
	1–3 times a month	29 (41.4%)	29 (39.7%)		12 (48.0%)	12 (54.6%)	
	Once a week	4 (5.7%)	6 (8.2%)		1 (4.0%)	2 (9.1%)	
	Several times a week	0 (0.0%)	1 (1.4%)		2 (8.0%)	1 (4.5%)	
	Once a day	0 (0.0%)	0 (0.0%)		0 (0.0%)	1 (4.5%)	
	Several times a day	0 (0.0%)	0 (0.0%)		0 (0.0%)	0 (0.0%)	
Energy drinks	Never	68 (97.1%)	70 (95.9%)	0.6836	22 (88.0%)	17 (77.3%)	0.3288
	1–3 times a month	2 (2.9%)	3 (4.1%)		3 (12.0%)	5 (22.7%)	
	Once a week	0 (0.0%)	0 (0.0%)		0 (0.0%)	0 (0.0%)	
	Several times a week	0 (0.0%)	0 (0.0%)		0 (0.0%)	0 (0.0%)	
	Once a day	0 (0.0%)	0 (0.0%)		0 (0.0%)	0 (0.0%)	
	Several times a day	0 (0.0%)	0 (0.0%)		0 (0.0%)	0 (0.0%)	
Water	Never	4 (5.7%)	1 (1.4%)	0.7349	1 (4.0%)	1 (4.6%)	0.5706
	1–3 times a month	2 (3.0%)	2 (2.7%)		1 (4.0%)	0 (0.0%)	
	Once a week	1 (1.4%)	1 (1.4%)		1 (4.0%)	1 (4.6%)	
	Several times a week	5 (7.1%)	8 (11.0%)		6 (24.0%)	2 (9.0%)	
	Once a day	5 (7.1%)	7 (9.5%)		0 (0.0%)	1 (4.6%)	
	Several times a day	53 (75.7%)	4 (74.0 %)		16 (64.0%)	17 (77.2%)	
Alcoholic drinks	Never	13 (18.6%)	23 (31.5%)	0.3729	4 (16.0%)	2 (9.1%)	0.2701
	1–3 times a month	33 (47.1%)	29 (39.7%)		5 (20.0%)	5 (22.7%)	
	Once a week	16 (22.9%)	18 (24.7%)		6 (24.0%)	3 (13.6%)	
	Several times a week	8 (11.4%)	3 (4.1%)		7 (28.0%)	11 (50.0%)	

Once a day	0 (0.0%)	0 (0.0%)	3 (12.0%)	0 (0.0%)
Several times a day	0 (0.0%)	0 (0.0%)	0 (0.0%)	1 (4.6%)

[1]Men NCF: $n = 24$

[2]Women NCF: $n = 69$

MCI—mild cognitive impairment; NCF—normal cognitive function
